# Mechanism of non-coding RNA regulation of DNMT3A

**DOI:** 10.1186/s13072-025-00574-w

**Published:** 2025-03-28

**Authors:** Jonathan E. Sandoval, Nancy V. N. Carullo, Aaron J. Salisbury, Jeremy J. Day, Norbert O. Reich

**Affiliations:** 1 Department of Molecular, Cellular and Developmental Biology, University of California, Santa Barbara, CA 93106-9510 USA; 2 Department of Chemistry and Biochemistry, University of California, Santa Barbara, CA 93106-9510 USA; 3 Department of Neurobiology, University of Alabama at Birmingham, Birmingham, AL 35294 USA

**Keywords:** DNA methyltransferase 3A (DNMT3A), Protein-RNA interactions, Epigenetics, DNA methylation, Allostery, DNMT3L, Cancer, Gene regulation

## Abstract

**Background:**

De novo DNA methylation by DNMT3A is a fundamental epigenetic modification for transcriptional regulation. Histone tails and regulatory proteins regulate DNMT3A, and the crosstalk between these epigenetic mechanisms ensures appropriate DNA methylation patterning. Based on findings showing that *Fos* ecRNA inhibits DNMT3A activity in neurons, we sought to characterize the contribution of this regulatory RNA in the modulation of DNMT3A in the presence of regulatory proteins and histone tails.

**Results:**

We show that *Fos* ecRNA and mRNA strongly correlate in primary cortical neurons on a single cell level and provide evidence that *Fos* ecRNA modulation of DNMT3A at these actively transcribed sites occurs in a sequence-independent manner. Further characterization of the *Fos* ecRNA-DNMT3A interaction showed that *Fos-1* ecRNA binds the DNMT3A tetramer interface and clinically relevant DNMT3A substitutions that disrupt the inhibition of DNMT3A activity by *Fos-1* ecRNA are restored by the formation of heterotetramers with DNMT3L. Lastly, using DNMT3L and *Fos* ecRNA in the presence of synthetic histone H3 tails or reconstituted polynucleosomes, we found that regulatory RNAs play dominant roles in the modulation of DNMT3A activity.

**Conclusion:**

Our results are consistent with a model for RNA regulation of DNMT3A that involves localized production of short RNAs binding to a nonspecific site on the protein, rather than formation of localized RNA/DNA structures. We propose that regulatory RNAs play a dominant role in the regulation of DNMT3A catalytic activity at sites with increased production of regulatory RNAs.

**Supplementary Information:**

The online version contains supplementary material available at 10.1186/s13072-025-00574-w.

## Background

The establishment of mammalian de novo DNA methylation patterns by the DNA methyltransferase 3A (DNMT3A) involves the modulation of DNMT3A activity by a wide range of biological molecules, including histone tails, regulatory proteins, and RNA [1–8]. Although there is a growing interest in understanding these regulatory mechanisms, few studies have identified specific interactions by which the crosstalk between de novo DNA methylation, histone modifications, regulatory proteins, and RNA translates into meaningful biological outcomes [9–11]. For instance, the loss of H3K4me2, H4 acetylation, and gain of H3K27me3 on one X chromosome attenuate the biallelic expression of *Tsix* RNA (antisense) and activate Xist expression (sense) at the onset of X chromosome inactivation [12]. The presence of H3K4me2, H4 acetylation and continual *Tsix* RNA expression on the other X chromosome sequester DNMT3A and activate DNMT3A activity to silence *Xist* expression [12]. In addition to playing a critical role in mammalian development, DNMT3A activity is linked to many aspects of neuronal function as well as memory formation and maintenance [13–16]. Furthermore, there is growing evidence that this role of DNMT3A in neurons, along with other epigenetic mechanisms, is influenced by regulatory RNAs in the central nervous system [17]. Savell et al. showed that extra-coding RNAs (ecRNAs), defined as non-polyadenylated RNAs originating from the sense strand of regions outside gene boundaries (transcription start and end sties), are critical modulators of DNMT3A activity in neurons [1]. More specifically, this occurs with FOS proto-oncogene ecRNA (*Fos* ecRNA), whose protein counterpart serves as a marker for neuronal activation [1, 18]. Furthermore, *Fos* ecRNA synthesis is increased by neuronal activation, leads to hypomethylation of the *Fos* gene through the direct inhibition of DNMT3A, and contributes to long-term fear memory formation in adult rats [1]. However, the underlying mechanism and extent of the modulation of DNMT3A activity by RNA generally, and *Fos* ecRNA in the presence of additional epigenetic mechanisms, like histone tails and regulatory proteins, remains unexplored.

Previous studies have demonstrated that distinct classes of regulatory RNAs, such as non-coding RNAs and microRNAs, play essential roles in the modulation of DNA methylation by directly associating with DNA methyltransferases (DNMTs) [2, 19, 20]. Furthermore, mechanistic studies of this modulation show that antisense CCAAT/enhancer-binding protein α (asCEBPA) RNA is a mixed inhibitor of DNMT1 and antisense *E-cadherin (CHD)* RNA is a non-competitive or mixed inhibitor of DNMT3A [2, 19]. While these studies on short regulatory RNAs (< 25 nucleotides) provide compelling evidence that RNA and DNA substrates bind to the same form of the enzyme, in addition to enzyme–DNA complexes, these findings are inconsistent with RNA binding to the active site of the enzyme and leave the surface on DNMT3A that binds regulatory RNAs uncharacterized. Furthermore, while the precise mechanism of RNA-induced inhibition *of* DNMTs in cells remains unknown, current models for the direct inhibition of DNMTs by RNAs in cells predict RNAs target DNMTs to specific genomic regions in a sequence specific manner [1, 21]. For example, the restricted expression observed in specific types of regulatory RNAs with short half-lives and expressed from enhancer regions suggests that regulatory RNAs may limit the modulation of DNMTs to local genomic microenvironments [21]. An alternative possibility, which stems from the prevalence of R-loops in DNA regulatory elements, is that regulatory RNAs form triplex structures by annealing to duplex DNA, provide a binding site for DNMTs and anchor DNMTs to specific loci [20, 22, 23]. The characterization of protein-RNA interactions has proven challenging due to the inherent flexibility of RNAs and the diversity of local RNA-binding surfaces on proteins [24]. However, structural analysis of the surfaces of proteins that bind both DNA and RNA reveal nucleic acid-specific differences in features like surface topology, solvent accessibility, and distribution of secondary structures [25]. For example, while the core domain of p53 (residues 98–292) binds DNA in a sequence‐specific manner, regulatory RNAs bind the C-terminal tetramerization domain (residues 300–393) with no known sequence specificity [26]. Intriguingly, the tetramerization domain of p53 enables the formation of p53 homotetramers and p53-DNMT3A heterotetramers [3, 27], thus suggesting that surfaces associated with protein–protein interactions may also be involved in protein-RNA interactions, which excludes the need for DNA-RNA sequence complementarity in RNA-mediated modulation of proteins that bind both DNA and RNA.

Given the emerging role of regulatory RNAs in diseases like Acute Myeloid Leukemia (AML) and Uterine corpus endometrial carcinoma* (*UCEC*)* [28, 29], two diseases that also display aberrant DNA methylation [30, 31], there is a growing interest in understanding the crosstalk among distinct epigenetic mechanisms. Previous work from our lab has shown that some proteins that regulate DNMT3A play a dominant role over histone N-terminal tails [32]. We sought to expand on these findings by providing insights into how RNA modulates DNMT3A in the presence of histone tails and regulatory proteins. For the latter, we relied on DNMT3L as an example of a partner protein whose physical interaction and functional perturbations of DNMT3A are well known [6, 33]. With the use of mutational mapping and biochemical assays consisting of DNMT3A, *Fos-1/-2* ecRNAs, DNMT3L and reconstituted polynucleosomes under various conditions*,* our data suggest that *Fos-1* ecRNA likely binds to the tetramer interface of DNMT3A and that these regulatory RNAs play a dominant role in modulating the enzymatic activity of DNMT3A in DNMT3A-Histone tail-DNMT3L- *Fos-1* ecRNA complexes. We also show *Fos* ecRNA accumulates at actively transcribed genomic regions of primary neurons and provide evidence that the modulation of DNMT3A activity by RNA transcripts does not rely entirely on sequence specificity, and contrary to the current models, inhibition of DNMTs by RNAs may involve transcripts that are nonspecific to the target gene [1, 20]. Lastly, we found that DNMT3L restores *Fos-1* ecRNA inhibition of substitutions in DNMT3A to residues that are correlated with AML and UCEC (R729, R736 and R771) and are differentially responsive to *Fos-1* ecRNA.

## Results

### Single molecule fluorescent in situ hybridization (smFISH) reveals correlation between Fos ecRNA and mRNA on a single cell level

**Fig. 1:**
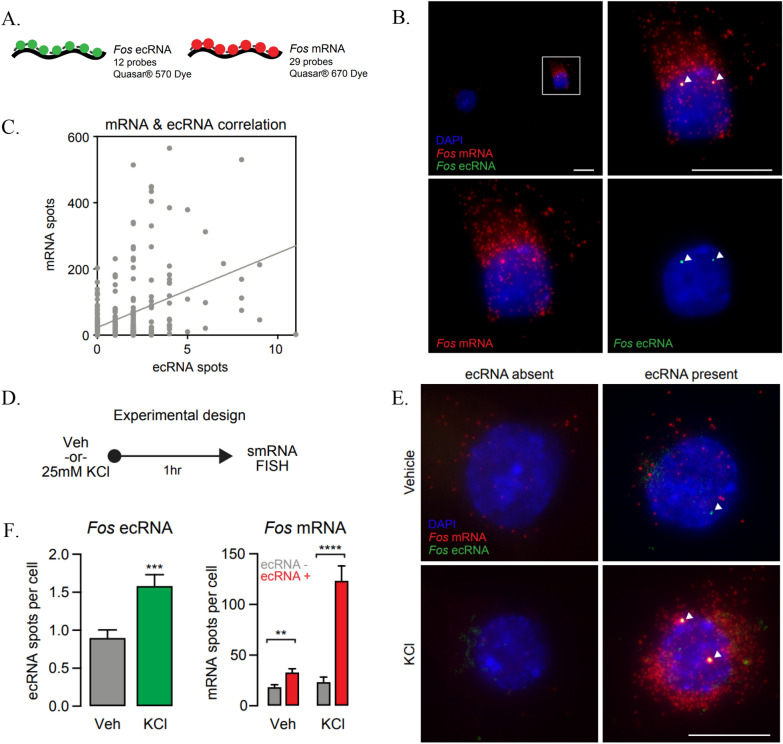
*Fos* ecRNA synthesis correlates with active transcription. **A** Illustration of smFISH probe sets indicating number of probes, dye, and LUT. **B** Representative smFISH images for *Fos* ecRNA (Quasar^®^ 570) and *Fos* mRNA (Quasar^®^ 670) transcripts. Cell nuclei are stained with DAPI (blue), RNA transcripts are marked by smFISH probes (red, green). Scale bar = 5 μm. **C** Comparison and correlation of detected *Fos* mRNA, and *Fos* ecRNA spots per cell reveal a significant positive correlation on a single cell level (Pearson correlation, R^2^ = 0.1869, p < 0.0001). **D** Experimental design for neuronal depolarization experiments. **E** Representative images of *Fos* ecRNA (Quasar^®^ 570) and *Fos* mRNA (Quasar^®^ 670) transcripts with or without neuronal depolarization. Cell nuclei are stained with DAPI (blue), RNA transcripts are marked by smFISH probes (red, green). Scale bar = 5 μm. **F** Summary data of ecRNA (left panel) and mRNA (tight panel) after 1 h of Veh or 25 mM KCl treatment demonstrate ecRNA levels increase in response to stimulation (unpaired t-test with Welch’s correction n(Veh) = 158, n(KCl) = 178, t = 3.725, p = 0.0002). Cells that express ecRNA express more *Fos* mRNA transcripts at baseline and in response to KCl (Multiple *t*-tests with fewer assumptions and Holm-Si-dak correction n(Veh) = 179, t = 3.35662, p = 0.000966, n(KCl) = 161, t = 5.58467, p < 0.000001). Data expressed as mean ± s.e.m. Multiple comparisons, *p < 0.05, **p < 0.01, ***p < 0.001, ****p < 0.0001

Previous studies show that the direct binding of *Fos-1* ecRNA to DNMT3A inhibits DNA methylation activity, results in hypomethylation of the *Fos* gene, and is required for the formation of long-term fear memories in rats [1]. To further understand how *Fos* ecRNA modulates de novo DNA methylation, we sought to gain insights into the distribution of ecRNAs and their response to KCl-mediated neuronal activation by performing single molecule fluorescent in situ hybridization (smFISH), a technique that allows visualization of individual ecRNA and mRNA transcripts on a single cell level. Using this tool, we investigated whether the number of RNA transcripts of interest changed in response to neuronal activation. Additionally, we investigated whether ecRNA and mRNA expression are correlated across individual neurons. Primary cortical neurons were depolarized with potassium chloride (KCl, 25 mM) for 1 h prior to fixation, permeabilization, and hybridization with fluorescently labeled smFISH probes. We designed custom probe sets to selectively target and mark individual *Fos* mRNA transcripts, as well as *Fos* ecRNA (Fig. 1A, B). To determine whether ecRNA and mRNA levels are correlated at the single-cell level, we multiplexed probe sets targeting *Fos* mRNA and *Fos* ecRNA. These experiments revealed a significant correlation of *Fos* ecRNA and *Fos* mRNA transcript numbers on a single cell level (Fig. 1C), with higher mRNA levels found in cells with more ecRNA foci.

As shown in Fig. 1D–F, the number of *Fos* ecRNA transcripts increased in response to KCl-mediated neuronal depolarization. It is possible that this effect is stronger than represented in our analysis due to overlapping signal in neurons with high transcript abundance (which would potentially cause overlapping or nearby spots to be counted together). Interestingly, we detected only a few, but very discrete puncta per cell with the ecRNA probe set. Larger high-intensity foci are typically associated with active transcription sites as they indicate an accumulation of transcripts. We observed this phenomenon frequently in the quantification of *Fos* mRNA signal, where active transcription is expected in response to depolarization. However, for ecRNAs, we found such high-intensity puncta to occur much more frequently in both treatment groups, suggesting an accumulation of transcripts at these sites. Notably, we also observed that KCl treatment only increased *Fos* mRNA counts in cells where *Fos* ecRNA was present, and neurons with ecRNA signal exhibited more *Fos* mRNA puncta in both vehicle and KCl-stimulated neurons. Taken together, these findings indicate that ecRNAs contribute to transcriptional regulation of their target genes not only on a cell population level but also on a single cell level.

### DNMT3A_CD tetramer interface mutants are differentially responsive to inhibition of enzymatic activity by Fos-1 ecRNA and inhibition does not require a DNA-Fos ecRNA complex

**Fig. 2:**
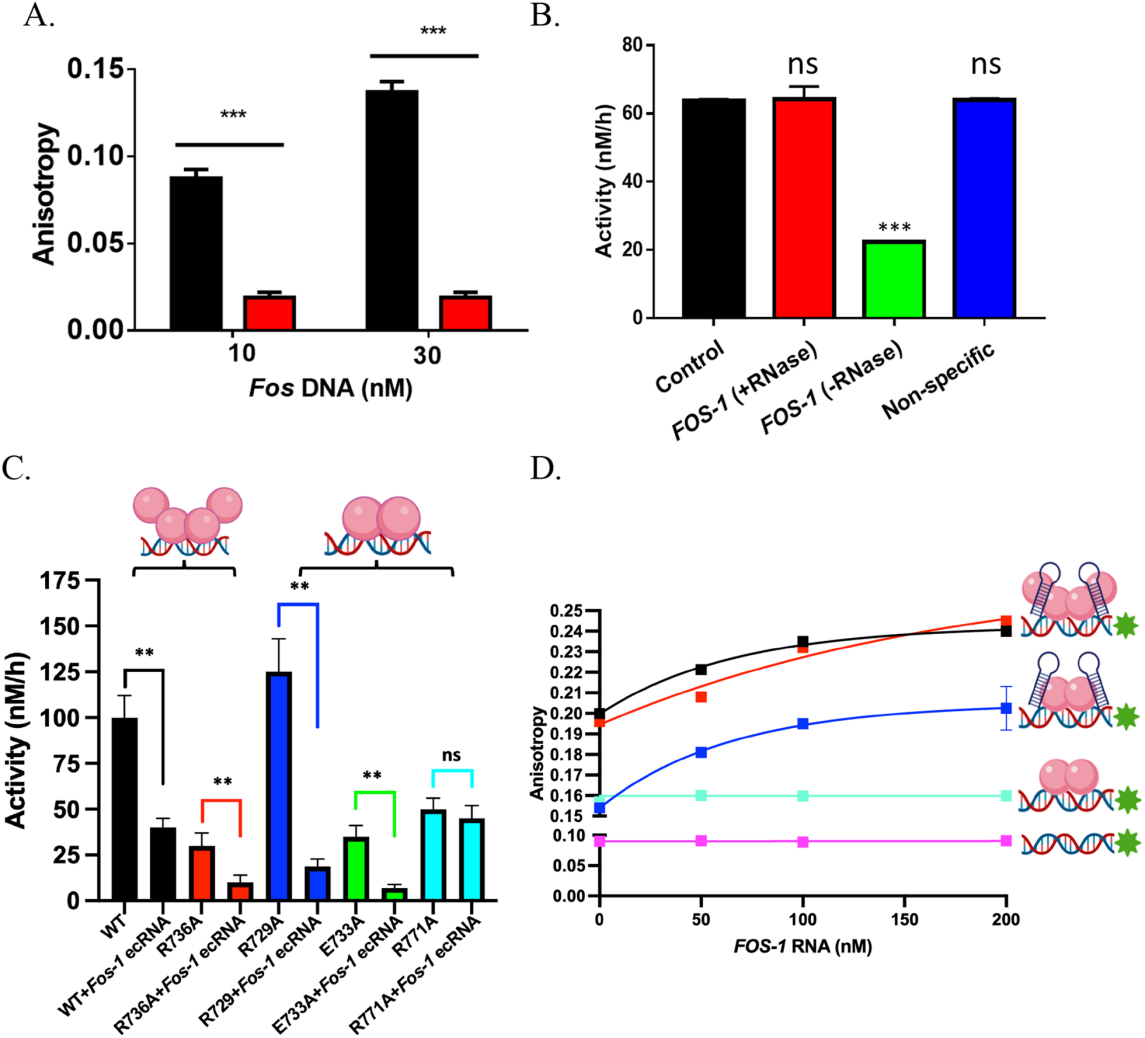
*Fos-1* ecRNA does not disrupt DNA-bound DNMT3A and inhibition requires binding to the DNMT3A tetramer interface. **A** The addition of *Fos* DNA (NCBI Gene ID 2353, 3′- 500 nucleotide duplex DNA of the *Fos* gene body) increases the fluorescence anisotropy of a 5′ FAM-6-labeled 18-mer positive control RNA () but not that of 5′ 6-FAM-labeled *Fos-1* ecRNA (). **B** Reactions consisting of () no RNA, () RNase treatment of *Fos-1* ecRNA prior to the start of the reaction, () *Fos-1* RNA and () a non-specific RNA show that *Fos-1* ecRNA specifically inhibits DNMT3A_CD^WT^ activity. **C** Radiochemical assays show DNMT3A_CD tetramer interface mutants are differentially responsive to inhibition of enzymatic activity by *Fos-1* ecRNA. **D** The addition of *Fos-1* ecRNA increases the fluorescence anisotropy of DNA-bound (10 nM of 5′ FAM-6-labeled GCbox30 duplex) () WT, () R736A and () R729A but not () R771A DNMT3A_CD enzymes (1 µM). A reaction with () *Fos-1* RNA in the presence of DNA was included as a control. Reactions in (**A**) consisted of 500 nM of each FAM-labelled RNA. All DNA methylation reactions, (**B**) with 50 nM enzyme and (**C**) with 150 nM enzyme, were initiated by the addition of Poly dI–dC (5 μM) only or a pre-mixture of RNA (1 μM) and Poly dI–dC (5 μM). Cartoon depiction of (**C**) and (**D**) depict the oligomeric state as previously characterized [41]. Data reflect the mean and standard deviation of 3 experiments. A one-way analysis of variance was used to compare values to a control (**B**) or of each mutant to wild type (**C**) (****p < 0.001; ***p < 0.001; ns, *p* > 0.05)

Work from our lab has provided insights into the mechanisms of RNA-mediated inhibition of DNMT3A using a wide-range of biologically significant RNA sequences [2]. However, the surface on DNMT3A that binds regulatory RNAs and how regulatory RNAs restrict DNMT3A function to a specific locus remain uncharacterized. To probe whether inhibition of DNMT3A by *Fos* ecRNA requires the formation of an ecRNA-DNA complex, we monitored the fluorescence anisotropy of a 5′ 6-FAM-labeled *Fos-1* ecRNA (5′-GGGGACACGCCCUCUGUUCCCUUAU-3′) as well as a 5′ 6-FAM-labeled 18-mer RNA designed to form a complex with the human *Fos* gene body (NCBI Gene ID 2353, 3′- 500 nucleotide duplex) (Fig. S1 A.) (Fig. 2A). We employed this segment of the *Fos* gene due to its proximity to the sites of *Fos* ecRNA synthesis [1]. Increasing concentrations of *Fos* DNA (10 or 30 nM) increases the fluorescence anisotropy of the 5′ 6-FAM-labeled 18-mer RNA (Fig. 2A ) but not of 5′ 6-FAM-labeled *Fos-1* ecRNA (Fig. 2A ), indicating that *Fos-1* ecRNA does not form a complex with this portion of the *Fos* gene. We previously used computational modeling and mutational mapping to implicate the tetramer interface of DNMT3A as a potential surface on DNMT3A for interactions with p53 [3], and in a similar manner, we employed a hybrid docking algorithm of template-based modeling and free docking to predict a surface on DNMT3A for interactions with *Fos-1* ecRNA [34]. *Fos-1* ecRNA displays comparable binding to the full-length and catalytic domain of DNMT3A [1]. In addition, the full-length and catalytic domain of DNMT3A display similar kinetic parameters (*k*_cat_, *K*_*m*_^DNA^, *K*_*m*_^AdoMet^), as well as modulation by distinct partner proteins [8, 32, 35, 36]. Based on these observations, we initially relied on the use of the catalytic domain of DNMT3A with subsequent experiments involving the full-length enzyme. We first sought to confirm that the previously reported inhibition by *Fos-1* ecRNA is specific to DNMT3A, and that this inhibition is not limited to the *Fos-1* gene as a substrate, as suggested by *Fos-1* ecRNA not forming a complex with *Fos* DNA (Fig. 2A). For this, we assessed *Fos-1* ecRNA inhibition on Poly dI-dC as a substrate, a commonly used DNA substrate for the study of DNA-modifying enzymes due to the large number of potential methylation sites [19, 37–40]. Consistent with previous findings, we found that reactions initiated by a mixture of *Fos-1* ecRNA (5′-GGGGACACGCCCUCUGUUCCCUUAU-3′) and Poly dI-dC as a DNA substrate (Fig. 2B ) displayed roughly a 50% decrease in DNMT3A_CD activity compared to reactions consisting of Poly dI-dC only (Fig. 2B ) [1]. Additionally, this decrease in DNA methylation by *Fos-1* ecRNA was observed in reactions consisting of DNMT3A_CD (Fig. S1B ) and DNMT1 (Fig. S1B ) but not M. HhaI (Fig. S1B ), a bacterial CpG methyltransferase. No inhibition to DNMT3A activity was observed in similar reactions involving a mixture of *Fos-1* ecRNA and Poly dI-dC that was treated with RNase prior to assaying for methylation activity (Fig. 2B ) or a mixture of Poly dI-dC and a non-specific RNA (5′-CGACCGCCUACUGAAAGAGGGC-3′) previously employed as a material for nanoparticle construction (Fig. 2B ) [47]. Furthermore, we observed that *Fos-2* ecRNA (5′-GUCUGUGCACCGUGUGCAUAUACAG-3′) (Fig. S1C ) is a more potent inhibitor of DNMT3A_CD activity compared to *Fos-1* ecRNA (Fig. S1C ). Given previous work by Savell et al. showing that modulation of DNMT3A activity by *Fos-1* ecRNA is essential for neuronal DNA methylation dynamics, we focused on this RNA molecule for further biochemical characterization of the interactions with DNMT3A [1].

We previously used alanine scanning to identify residues on the DNMT3A tetramer interface that largely contribute to the formation of higher order complexes and docking-based modeling of protein–protein interfaces to predict a surface on DNMT3A for interactions with p53 [3, 41]. In a similar manner, previous studies have relied on a hybrid docking algorithm of template-based modeling and free docking (HDOCK server) to predict protein surfaces for protein-RNA interactions [34, 42–44]. Using the monomeric form of DNMT3A (PDB: 5YX2; residues 628–914) and the *Fos-1* ecRNA sequence, we relied on this approach to predict a surface on DNMT3A for interactions with *Fos-1* ecRNA [34, 45]. Computational models generated in the HDOCK server were used to predict the DNMT3A tetramer interface as a likely surface for DNMT3A- *Fos-1* ecRNA interactions (Fig. S2). Based on these predictions, we assessed *Fos-1* ecRNA inhibition of DNMT3A activity in a subset of alanine substitutions to residues at the DNMT3A tetramer interface, which vary in their oligomeric state (R729A, E733A, R736A, R771A) [41]. Interestingly, substitutions to these residues at the tetramer interface of DNMT3A are frequently observed in AML or UCEC patients (TCGA) [46, 49]. We observed that although the extent of *Fos-1* ecRNA inhibition varied across the substitutions examined (Fig. 2C, () DNMT3A_CD^R736A^, () DNMT3A_CD^R729A^ and () DNMT3A_CD^E733A^), DNMT3A_CD^R771A^ (Fig. 2C ) was the only substitution that displayed no inhibition. We then sought to assess whether the lack of *Fos-1* ecRNA inhibition of DNMT3A_CD^R771A^ is due to the inability of DNMT3A_CD^R771A^ to bind *Fos-1* ecRNA (Fig. 2D) by monitoring the fluorescence anisotropy of DNMT3A complexes on DNA, an approach previously employed to monitor interactions at the DNMT3A tetramer interface [3]. The addition of *Fos-1* ecRNA resulted in a corresponding increase to the initial anisotropy of DNA-bound DNMT3A_CD^WT^ (Fig. 2D ), DNMT3A_CD^R736A^ (Fig. 2D ), DNMT3A_CD^R729A^ (Fig. 2D ) but not of DNMT3A_CD^R771A^ (Fig. 2D ) or DNA only (Fig. 2D ). Thus, showing that the addition of *Fos-1* ecRNA to DNA-bound DNMT3A_CD^R771A^ does not lead to the formation of higher order complexes on DNA (Fig. 2D ) and that *Fos-1* ecRNA inhibition (Fig. 2C) does not stem from disrupting DNA-bound DNMT3A (Fig. 2D WT , R736A , R736A ). The tetramer interface of DNMT3A is well characterized as is the regulation of DNMT3A activity by a wide range of regulatory proteins with distinct functional outcomes [3–5]. Our results suggest that modulation of DNMT3A activity by RNA may also occur through interactions with residues at the tetramer interface of DNMT3A and it is not likely relying on the formation of a DNA-*Fos* ecRNA complex (Fig. 6A).

### The oligomeric state of DNMT3A_CD affects the mechanism of allosteric inhibition by Fos-1 ecRNA

**Fig. 3:**
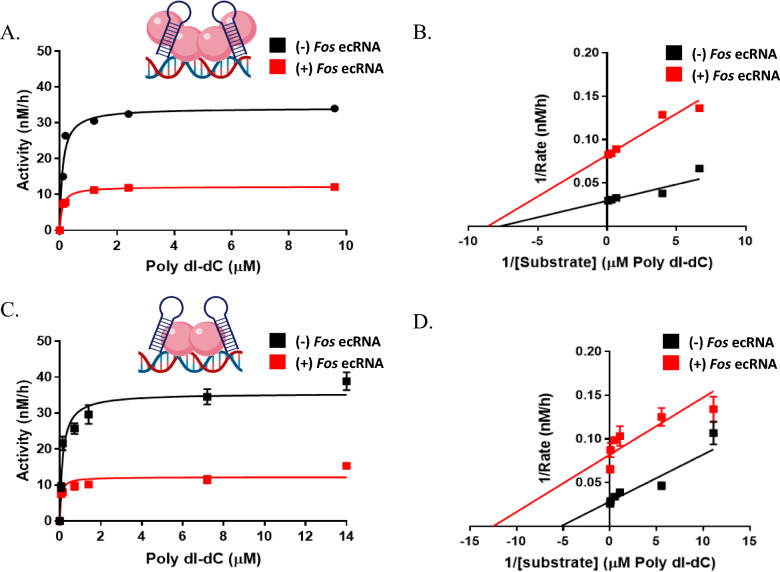
The oligomeric state of DNMT3A_CD affects modulation of enzymatic activity by *Fos-1* RNA. Velocity curves of (**A**) DNMT3A_CD^WT^ homotetramers or (**C**) DNMT3A_CD^R729A^ homodimers in the absence () or presence of excess *Fos-1* ecRNA (1 µM) with increasing concentration of Poly dI–dC as a DNA substrate (). **B** and **D** double reciprocal plots for (**A**) and (**C**), respectively. In (**A**) and (**C**), reactions consisting of 30 nM enzymes were initiated by the addition of a pre-mixture of *Fos-1* ecRNA (1 μM) and Poly dI-dC (5 μM). Data reflect the mean and standard deviation of 3 experiments

Studies aiming to probe the mechanism of DNMT1 inhibition by asCEBPA suggest that this short non-coding RNA (23 nucleotides) is a mixed inhibitor of DNMT1; thus, inhibition may occur through the direct binding of asCEBPA to DNMT1 or to the DNMT1–hemimethylated DNA complex [19]. Similarly, mechanism of inhibition studies involving DNMT3A and *CHD* RNA support non-competitive or mixed type models [2]. We previously showed that the oligomeric state of DNMT3A tetramer interface mutants affects processive catalysis and modulation by distinct partner proteins [3, 4, 47]. Given that DNMT3A_CD tetramer interface mutants are differentially responsive to *Fos-1* ecRNA inhibition relative to DNMT3A_CD^WT^ (Fig. 2B), we sought to assess whether the altered oligomeric state of DNMT3A_CD tetramer interface mutants influences the mechanism of inhibition by *Fos-1* ecRNA. In addition, we sought to examine whether *Fos-1* ecRNA-mediated inhibition of DNMT3A_CD derives from direct *Fos-1* ecRNA competition with substrate DNA binding to the active site of DNMT3A_CD. For this approach, we carried out methylation assays with varying DNA concentrations and saturating RNA (Fig. S1 C.) using a dimeric DNMT3A_CD tetramer interface mutant (R729A) that is responsive to *Fos-1* ecRNA inhibition (Fig. 2B). While the addition of *Fos-1* ecRNA to DNMT3A_CD^WT^ led to a reduced V_MAX_ but did not affect *K*_M_ (Fig. 3A and B ), the addition of *Fos-1* ecRNA led to reduced *K*_M_ and V_MAX_ in reactions consisting of DNMT3A_CD^R729A^ (Fig. 3C and D ). Thus, the inhibition data for DNMT3A_CD^WT^ (Fig. 3A and B ) best fits a noncompetitive model while the results for DNMT3A_CD^R729A^ (Fig. 3C and D ) are more consistent with an uncompetitive model. Our results show the oligomeric state of DNMT3A affects inhibition by *Fos-1* ecRNA and exclude any mechanism that invokes competition with substrate DNA. Therefore, our data supports an allosteric mechanism of inhibition (Fig. 3).

### DNMT3L restores inhibition of DNMT3A_CD^R771A^ by Fos-1 ecRNA

**Fig. 4:**
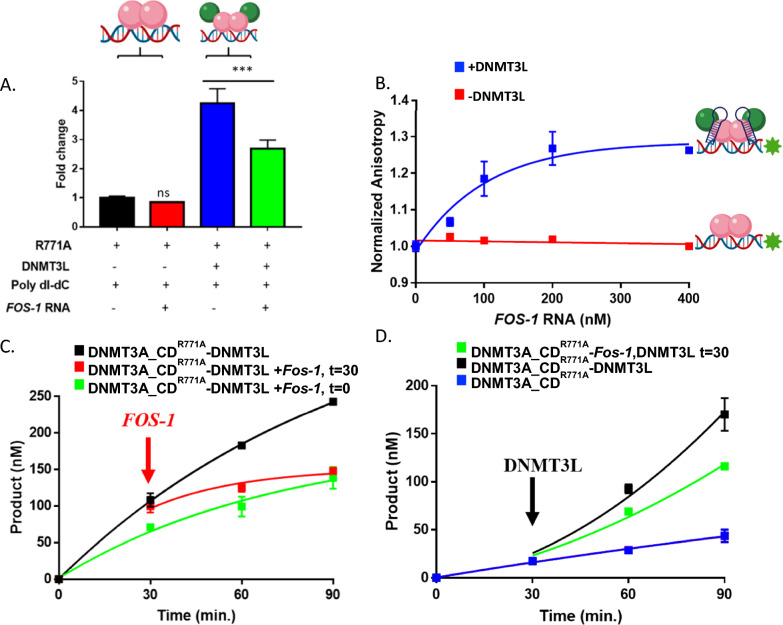
Formation of DNMT3A_CD^R771A^ heterotetramers with DNMT3L restores inhibition of enzymatic activity by *Fos-1* ecRNA. **A** Although *Fos-1* ecRNA does not inhibit DNMT3A_CD ^R771A^ homodimers (), the addition of *Fos-1* ecRNA leads to the dominant inhibition of DNMT3A_CD^R771A^**-**DNMT3L heterotetramers (). All reactions in (**A**) consisted of proteinss at 150 nM (1:1 to DNMT3A tetramer) and were initiated by the addition of Poly dI-dC (5 μM) or a pre-mixture of *Fos-1* ecRNA (1 μM) with Poly dI-dC (5 μM). **B** The addition of *Fos-1* ecRNA leads to an increase in the fluorescence anisotropy of DNA-bound (10 nM of 5′ FAM-6-labeled GCbox30 duplex) DNMT3A_CD^R771A^**-**DNMT3L heterotetramers () but not DNMT3A_CD^R771A^ homodimers (). Data are normalized to initial anisotropy values of DNA-bound DNMT3A_CD^R771A^. **C** *Fos-1* ecRNA disrupts actively catalyzing DNMT3A_CD^R771A^**-**DNMT3L heterotetramers (). **D** Reactions with DNMT3A_CD^R771A^ initiated by the addition of a pre-mixture of *Fos-1* ecRNA and Poly dI-dC challenged with the addition of DNMT3L () display reduced activity relative to similar reactions but in the absence of *Fos-1* ecRNA (). The following reactions were performed as controls: DNMT3A_CD^R771A^**-**DNMT3L with Poly dI-dC only (**C**), DNMT3A_CD^R771A^**-**DNMT3L with *Fos-1* ecRNA and Poly dI-dC at the start of the reaction (**C**), or DNMT3A_CD^R771A^ with Poly dI-dC only (**D**). Data reflect the mean and standard deviation of 3 experiments. In A., a one-way analysis of variance was used to compare values to DNMT3A_CD^R771A^ () (***p < 0.001; ns, *p* > 0.05)

DNMT3L, the inactive homolog of DNMT3A, serves as a stimulatory factor of de novo methylation by DNMT3A and is essential for the establishment of appropriate methylation patterns of maternally imprinted genes [33]. Furthermore, tetramerization of DNMT3A dimer mutants with DNMT3L restores processive catalysis [41, 47]**.** Based on this evidence, we sought to examine whether formation of DNMT3A_CD^R771A^-DNMT3L heterotetramers restores inhibition of enzymatic activity by *Fos-1* ecRNA as DNMT3L provides a well-studied model system for how partner proteins regulate the activity of DNMT3A mutants with altered oligomeric states (Fig. 2C and D). Initial reactions show *Fos-1* ecRNA decreases the DNA methylation activity of DNMT3A_CD^WT^-DNMT3L heterotetramers (Fig. S3 ) compared to similar reactions that did not include *Fos-1* ecRNA (Fig. S3 ). Surprisingly, although DNMT3A_CD ^R771A^ homodimers are unresponsive to the modulatory effect of *Fos-1* ecRNA (Fig. 4A  and ), we found that *Fos-1* ecRNA inhibits the activity of DNMT3A_CD^R771A^-DNMT3L heterotetramers (Fig. 4A  and ). We then assessed whether the observed inhibition (Fig. 4A  and ) is due to DNMT3A_CD^R771A^-DNMT3L heterotetramers binding *Fos-1* ecRNA by monitoring the fluorescence anisotropy of DNA-bound (GCbox30) DNMT3A_CD^R771A^ homodimers or DNMT3A_CD^R771A^**-**DNMT3L heterotetramers with increasing levels of *Fos-1* ecRNA (Fig. 4B). While the addition of *Fos-1* ecRNA consistently did not result in a detectable change to the initial anisotropy value of DNMT3A_CD^R771A^ homodimers (Fig. 2D  and Fig. 4B ), the titration of *Fos-1* ecRNA led to a corresponding increase to the initial anisotropy value of DNA-bound of DNMT3A_CD^R771A^**-**DNMT3L heterotetramers (Fig. 4B ). To better model the cellular dynamics between DNMT3A mutants, regulatory proteins and RNAs**,** we examined whether *Fos-1* ecRNA inhibition of DNMT3A_CD^R771A^-DNMT3L heterotetramers in equilibrium reactions (Fig. 4A) persists in actively catalyzing protein complexes (Fig. 4C and D). We show that the addition of *Fos-1* ecRNA (Fig. 4 C. ) disrupts the activity of DNMT3A_CD^R771A^-DNMT3L on DNA (Fig. 4C ). We also found that when challenged by the addition of DNMT3L, reactions consisting of DNMT3A_CD^R771A^ initiated by the addition of a pre-mixture of *Fos-1* ecRNA and Poly dI-dC (Fig. 4D ) are less catalytically active than similar reactions lacking *Fos-1* ecRNA (Fig. 4D ). In sum, our findings indicate that the formation of DNMT3A_CD^R771A^ heterotetramers with DNMT3L restores the ability of *Fos-1* ecRNA to inhibit the methylation activity of DNMT3A_CD^R771A^.

### Modulation of DNMT3A_FL^WT^ activity by Fos-1 ecRNA is dominant in the presence of histone H3 tails and DNMT3L

**Fig. 5:**
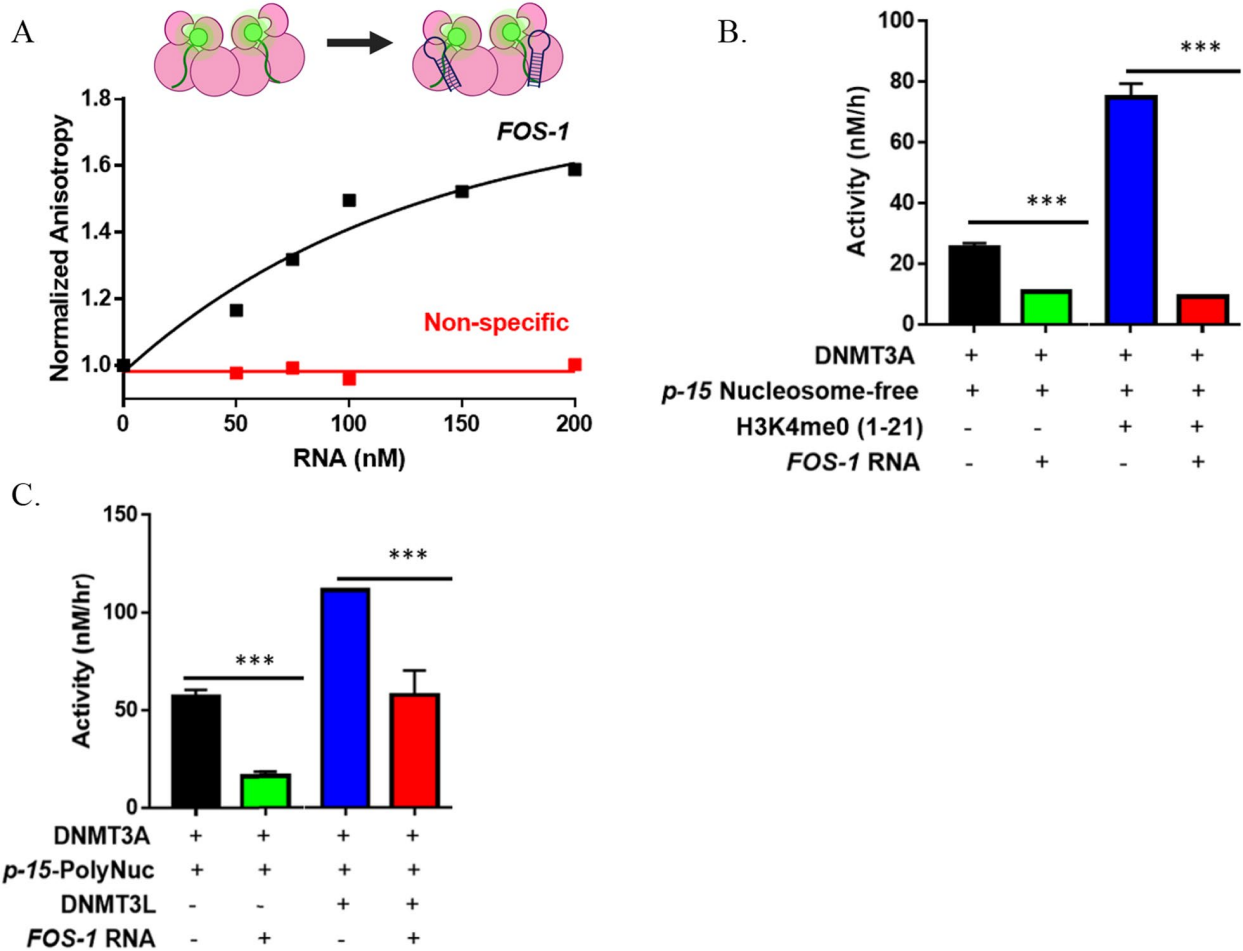
Modulation of DNMT3A enzymatic activity by RNA is dominant in DNMT3A_FL^WT^—DNMT3L- H3 tail- RNA complexes. **A** The fluorescence anisotropy of DNMT3A_FL^WT^ (1 μM) in complex with FAM-labeled H3K4me0 (residues 1–21; 10 nM) increases with the addition of *Fos-1* ecRNA () but not of non-specific RNA (). Data are normalized to initial anisotropy values of FAM-labelled H3- DNMT3A_FL^WT^ complexes. **B** *Fos-1* () ecRNA (1 μM) in the presence of H3K4me0 peptide (5 μM) disrupts stimulation of DNMT3A_FL^WT^ (50 nM) activity by H3K4me0 peptide () with *nucleosome-free p15 as a DNA substrate (*14 μM). Similar reactions consisting of *Fos-1* () ecRNA but in the absence of H3K4me0 peptide were performed as controls. **C** Excess *Fos-1* () ecRNA (30 μM; see Fig. S5) inhibits DNMT3A_FL^WT^ (150 nM) activity using p15 assembled into polynucleosomes (HeLa core histones) as a DNA substrate* (*14 μM)*.* Furthermore*,* inhibition of DNMT3A_FL^WT^ activity by *Fos-1* () ecRNAs is dominant in similar reactions containing DNMT3L 150 nM (1:1 to DNMT3A_FL^WT^ tetramer). DNMT3A was pre-incubated in reaction buffer for 1 h at 37 °C with H3K4me0 peptide (**B**) or DNMT3L (**C**) prior to reactions being initiated. DNA methylation reactions (**B** and **C**) were initiated by the addition of *p15* (Nucleosome free or polynucleosomes) or a pre-mixture of RNA (*Fos-1*) with *p15* (Nucleosome free or polynucleosomes). Data reflect the mean and standard deviation of 3 experiments. In B. and C., a one-way analysis of variance was used to compare values the values of reactions with *Fos-1* ecRNA to those of DNMT3A only or DNMT3A with H3K4me0 (**B**) or DNMT3L (**C**) (***p < 0.001; ns, *p* > 0.05)

**Fig. 6:**
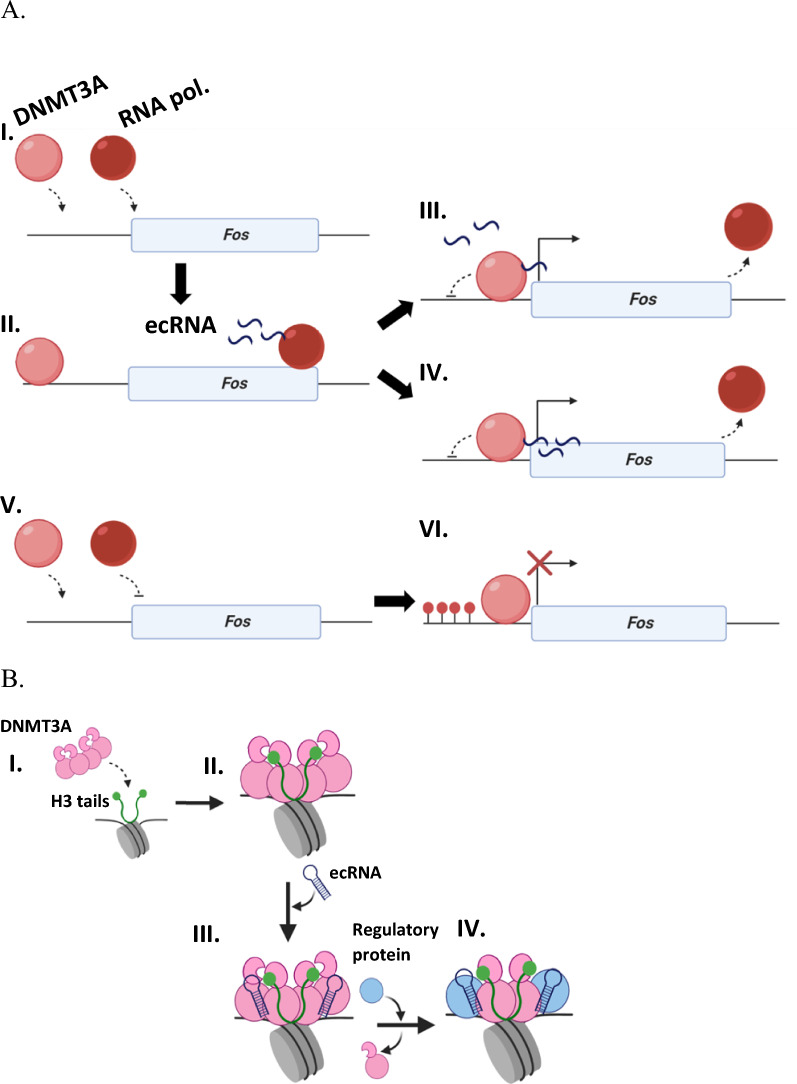
Models for ecRNA modulation of DNMT3A and interactions with additional components of the epigenetic machinery. **A** The synthesis of ecRNA (; II.) by RNA polymerase () can directly inhibit DNMT3A () through the absence (III.) or presence (IV.) of an ecRNA-DNA complex. In the absence of ecRNA synthesis (V.), DNMT3A () is not inhibited and methylates target sites (VI.; represented by red lollipops). (**B**) Following the localization of DNMT3A () by histone tails () to specific genomic loci (I. and II.), ecRNA () can access and dominantly modulate the activity of DNMT3A homo- (III.) or heterotetramers (IV.)

In addition to modulating cellular activity of DNMT3A [1, 2], distinct classes of non-coding RNAs contribute to epigenetic gene regulation by directly and indirectly modulating the enzymatic activities of histone modifying enzymes [48–51]. Based on this evidence, there is growing interest in understanding the role regulatory non-coding RNAs play in epigenetic control [11, 48]. However, much less is known about the crosstalk between non-coding RNAs, regulatory proteins and histone tails in the modulation of epigenetic enzymes. In the context of this crosstalk, we have shown that some regulatory proteins play a dominant role over histone N-terminal tails in the simultaneous modulation of DNMT3A [32]. Given that DNMT3A activity is modulated through the direct interactions with non-coding RNAs, histone N-Terminal tails*,* and a wide range of regulatory proteins, we sought to expand on our findings by assessing the modulation of DNMT3A activity by *Fos-1* ecRNA in the presence of histone tails, DNMT3L and using the *cyclin-dependent kinase inhibitor 2B* (*CDKN2B or p15*) gene promoter incorporated into a plasmid lacking any CpG sites (*p15-pCpG*^*L*^) [1–8]. We used the *p15-pCpG*^*L*^ as a substrate in both nucleosome free and *p15-pCpG*^*L*^ assembled into polynucleosomes as *p15-pCpG*^*L*^ is a well-established target of DNMT3A that has been previously characterized biochemically [4]. We relied on the use of DNMT3L as it provides a suitable model to study the simultaneous modulation of DNMT3A due to its well-characterized interactions with DNMT3A and predicted shared binding surface with *Fos-1* ecRNA (Fig. 2, Fig. S2) [6, 33, 41, 47]. Additionally, we used the H3K4me0 peptide [10], a potent activator of the enzymatic activity of DNMT3A, to detect any changes to the activation of DNMT3A by H3K4me0 in the presence of *Fos-1* ecRNA [52]. Experiments involving peptides derived from the human H3 tail were carried out using the full-length DNMT3A (DNMT3A_FL^WT^) which contains the Atrx-Dnmt3-Dnmt3l (ADD) domain necessary to bind histone tails, in addition to displaying similar kinetic parameters as the DNMT3A catalytic domain [8, 35]. Although our results indicate that *Fos-1* ecRNA and histone N-Terminal tails bind a distinct surface on DNMT3A (Fig. 2 and Fig. S2) [6–8], we assessed whether DNMT3A simultaneously accommodates *Fos-1* ecRNA and histone H3 tails by monitoring the fluorescence anisotropy of DNMT3A bound to FAM-labeled H3K4me0 peptide (residues 1–21; Fig. 5A; Fig. S4). While the addition of non-specific RNA did not lead to detectable changes in anisotropy of DNMT3A_FL^WT^- FAM-labeled H3K4me0 peptide at maximum anisotropy (Fig. 5A ), the addition of *Fos-1* ecRNA led to an increase to the initial anisotropy values of DNMT3A_FL^WT^- FAM-labeled H3K4me0 peptide (Fig. 5A ). Thus, *Fos-1* ecRNA can access DNMT3A in complex with histone H3 tails. Based on this finding, we then determined the functional consequences of a DNMT3A_FL^WT^-H3K4me0 peptide- *Fos-1* ecRNA complex on DNMT3A_FL^WT^ activity (Fig. 5B). Initial controls show that while the presence of *Fos-1* ecRNA (Fig. 5B ) reduces the activity of a DNMT3A_FL^WT^ on of *p15* (nucleosome free) as a DNA substrate, formation of DNMT3A_FL^WT^-H3K4me0 peptide complexes activates the enzymatic activity of DNMT3A (Fig. 5B ), as previously reported [52]. Interestingly, the activation of DNMT3A_FL^WT^ activity by H3K4me0 peptide (Fig. 5 B. ) was disrupted in reactions initiated by a pre-mixture of *Fos-1* ecRNA (Fig. 5B ) with *p15* (Nucleosome free). Thus, modulation of DNMT3A_FL^WT^ methylation activity by *Fos-1* ecRNA is dominant in DNMT3A_FL^WT^-*Fos-1* ecRNA -H3 tail complexes. To better approximate the simultaneous modulation of DNMT3A activity within cells, we then assessed the functional outcomes of DNMT3A_FL^WT^-*Fos-1* ecRNA -H3 tail complexes using *p15* assembled into polynucleosomes consisting of histone core proteins extracted from HeLa cells (Fig. 5C). We show that while 1 μM of *Fos-1* ecRNA sufficiently inhibits the enzymatic activity of DNMT3A_FL^WT^ on *p15* DNA (14 μM; Nucleosome free) (Fig. 5B; Fig. S5A), inhibition of DNMT3A_FL^WT^ with *p15* assembled into polynucleosomes* (*14 μM) *as a substrate* requires an excess concentration of *Fos-1* (Fig. 5C ) ec*RNAs* (Fig. S5 A. and B.). Furthermore, reactions initiated by a pre-mixture of 30 μM *Fos-1* ecRNA (Fig. 5C ) with *p15* assembled into polynucleosomes resulted in decreased activity compared to that of DNMT3A_FL^WT^-DNMT3*L* complexes using a similar substrate and protein concentrations (Fig. 5C ). However, the enzymatic activity of DNMT3A_FL^WT^-DNMT3L heterotetramers in the presence of *Fos-1* ecRNA (Fig. 5C ) was higher than that of similar reactions consisting of DNMT3A_FL^WT^ homotetramers and *Fos-1* ecRNA (Fig. 5C ) with *p15* assembled into polynucleosomes as a substrate*.* Under these conditions, we show that modulation of DNMT3A methylation activity by regulatory RNAs is dominant in DNMT3A- histone H3 tails-regulatory protein-RNA complexes (Fig. 6B).

## Discussion

The modulation of DNMT3A activity in the establishment of appropriate genomic DNA methylation patterns calls for the simultaneous association with a wide range of biological molecules, yet few studies have focused on the crosstalk between DNMT3A, histone modifications, regulatory proteins and RNAs; rather, most studies have largely focused on the individual effects of these modulators of DNMT3A [1–11]. While the interactions between DNMT3A and its modulators play a multifaceted role in embryonic development, oncogenesis, and neuronal function [13–16, 51], recent work linking *Fos* ecRNA-mediated inhibition of DNMT3A activity in neurons to fear memory formation further highlight the role of regulatory RNAs in the control of epigenetic mechanisms involved in cognitive function [1, 9, 12, 27, 33]. Based on these observations, along with the emerging role of regulatory RNAs as key modulators of the epigenetic machinery in a wide range of biological processes [1, 2, 11, 17, 28, 29, 48–53], we sought to characterize the dynamics and relative role of histone tails, regulatory proteins, and RNA in the simultaneous coordination of DNMT3A activity. To further explore the cellular and structural basis of *Fos-1* ecRNA-DNMT3A interactions, we also assessed the distribution of ecRNA in primary neurons in response to stimuli on a single-cell level and aimed to determine the surface on DNMT3A for interactions with *Fos-1* ecRNA. Our results are consistent with DNMT3A simultaneously accommodating H3 tails, regulatory proteins, and RNAs, and that regulatory RNAs play a dominant role in the modulation of DNMT3A methylation activity in these multiprotein-RNA complexes. We also show *Fos* ecRNA synthesis correlates with active transcription sites in primary neurons and provide evidence that the modulation of DNMT3A activity by ecRNA is not restricted to target sites through the formation of DNA-ecRNA complexes (Fig. 6A comparison of models III and IV). Furthermore, we found that *Fos-1* ecRNA binds to the tetramer interface of DNMT3A. Although substitutions to clinically relevant residues at this interface, such as R771A homodimers, are differentially responsive to *Fos-1* ecRNA, formation of hetero-tetramers between DNMT3A and partner proteins such as DNMT3L appears to restore the inhibition of enzymatic activity by *Fos-1* ecRNA. These findings provide insights into the respective roles and interplay between RNA- and protein-based regulatory mechanisms of DNMT3A and elucidate a molecular basis for how these interactions contribute to the epigenetic control of gene expression.

RNA-binding proteins comprise as much as 13% of the eukaryotic proteome and are involved in a wide-range of biological processes including DNA repair, transcriptional and translational regulation [54–56]. Despite the obvious biological importance of RNA-binding proteins, the mechanisms that underpin protein-RNA interactions remain obscure largely due to the challenges associated with the characterization of protein-RNA complexes, such as the conformational flexibility of RNA and structural diversity of RNA-binding surfaces on proteins [24, 57, 58]. However, previous work from our lab has provided insights into how distinct classes of regulatory RNAs modulate DNMT3A and suggests these RNAs may bind a surface on DNMT3A outside the active site, although the precise surface on DNMT3A that binds regulatory RNAs and how DNMT3A function is restricted to a specific locus remain uncharacterized [2]. Based on previous findings in distinct biological contexts, it has been proposed that the gene-specific modulation of DNMT3A by *Fos* ecRNA occurs in a DNA-RNA complex dependent or independent manner (Fig. 6A, III or IV) [1, 20, 23]. We show *Fos* ecRNA synthesis correlates with sites with active *Fos* transcription on a single-cell level (Fig. 1) and provide evidence that interactions between *Fos* ecRNA and DNA are not likely contributing to the modulation of DNMT3A by *Fos* ecRNA at a specific locus (Fig. 2A) (Fig. 6A, III). In addition to our oligonucleotide binding assay (Fig. 2A), the use of regulatory RNAs that are unrelated to DNA substrate sequences in our methylation assays further suggests that the synthesis of regulatory RNAs in actively transcribed regions is a main contributor to the site-specific modulation of DNMT3A (Fig. 6A).

We then sought to characterize the surface on DNMT3A that binds *Fos-1* ecRNA to better understand the interactions between DNMT3A and *Fos* ecRNA following the synthesis of ecRNA. Using a hybrid docking algorithm to generate computational models of DNMT3A monomers (PDB 4U7T; residues 468-912) and *Fos-1* ecRNA, we identified the tetramer interface on DNMT3A as a potential surface on DNMT3A involved with DNMT3A-*Fos-1* ecRNA interactions (Fig. S2) [6, 34, 42–44]. We found that mutations at the tetramer interface disrupt binding and inhibition of DNA methylation activity by *Fos-1* ecRNA, indicating that interactions between *Fos-1* ecRNA and key residues at this interface, such as R771, are essential for the formation of a DNMT3A- *Fos-1* ecRNA complex on DNA and modulation of DNMT3A activity (Fig. 2C and E). However, in the absence of additional structural information, we are unable to determine the precise residues involved in the DNMT3A- *Fos-1* ecRNA interaction.

The tetramer interface of DNMT3A is a well-characterized surface on DNTM3A for the direct binding of distinct regulatory proteins to modulate essential biological functions of DNMT3A, such as kinetic parameters and cellular localization [3–5, 32, 41, 59]. Our findings highlight the importance and versatility of the tetramer interface of DNMT3A by identifying a potential novel interacting partner of this surface of DNMT3A, which suggests that *Fos-1* ecRNA does not inhibit DNMT3A through direct competition with substrate DNA. We challenged this notion by probing the mechanism of inhibition by *Fos-1* ecRNA and additionally examined whether changes to the oligomeric state of DNMT3A, stemming from mutations at the tetramer interface, affect inhibition (Fig. 3) [47]. We found that while *Fos-1* ecRNA does not compete with substrate DNA in DNMT3A homotetramers (Fig. 3A and B) or homodimers (Fig. 3C and D), the oligomeric state of DNMT3A affects the mechanism of allosteric inhibition. Previous work from our lab shows that disruption of the oligomeric state of DNMT3A, due to mutations at the tetramer interface, additionally disrupt processive catalysis and that heterotetramerization of DNMT3A dimers with DNMT3L restores processivity [41, 47]. Based on this observation and the apparent importance of the DNMT3A tetramer interface for interactions with *Fos-1* ecRNA (Figs. 2B–E. and 3), we assessed whether DNMT3L rescues modulation of DNMT3A_CD^R771A^ by *Fos-1* ecRNA (Fig. 2C and E). Like that observed in the context of processivity [37, 50], we show that heterotetramerization of DNMT3A_CD^R771A^ with DNMT3L restores *Fos-1* ecRNA binding (Fig. 4B) and inhibition of DNMT3A_CD^R771A^ under distinct catalytic conditions (Fig. 4A, C, and D). Furthermore, our mutational analysis shows that *Fos-1* ecRNA can inhibit both tetramer (R736A) and dimer (R729A and E733) mutants (Fig. 2C). Taken together with our findings for DNMT3A_CD^R771A^ homo- (Fig. 2C and E) and heterotetramers (Fig. 4), we show that *Fos-1* ecRNA can inhibit DNMT3A found in distinct oligomeric states and that this inhibition requires the interactions of key residues at the tetramer interface, such as R771. Analysis of the DNMT3A-DMNT3L co-crystal structure shows that R771 of DNMT3A may interact with T225 and D226 T227 of DNMT3L [45]. Interestingly, a high propensity of T and D residues interactions with guanine are observed in protein-RNA complexes [58]. DNMT3L interactions with *Fos-1* ecRNA, which has a 60% GC content, may accommodate *Fos-1* ecRNA at the DNMT3A-DNMT3L tetramer interface in the absence of R771 to inhibit the catalytic activity of DNMT3A_CD^R771A^ by-DNMT3L heterotetramers (Fig. 4. Fig. S7). We propose a model for a DNMT3A- *Fos-1* ecRNA complex that is consistent with previous findings showing p53 binds regulatory RNAs at a surface associated with protein–protein interactions [3, 26, 27, 60]. Although H3 tails and p53 or TDG bind distinct allosteric sites on DNMT3A, we have previously shown that these regulatory proteins are dominant in the simultaneous modulation of DNMT3A activity [32]. We show here that while *Fos-1* ecRNA binding to the DNMT3A tetramer interface does not perturb interactions between H3 tails and DNMT3A (Fig. 5A), *Fos-1* ecRNA disrupts activation of DNMT3A activity by H3K4me0 (residues 1–21) (Fig. 5B) or DNMT3L in reactions with polynucleosome substrates (Fig. 5C). In sum, our results are inconsistent with models of RNA-mediated regulation of DNA methylation that requires some type of RNA–DNA hybridization and strongly supports a model in which DNA methylation changes result from allosteric binding of locally synthesized ecRNA to DNMT3A (Fig. 2A) [1, 20–23]. What remains intriguing is that while the disruption of *Fos* ecRNA secondary structure does not disrupt binding to DNMT3A [1], a non-specific RNA of 22 nucleotides in length does not regulate DNMT3A activity (Fig. 2B). This could be reconciled by the site on DNMT3A that binds short RNA is largely non-specific, but the functional perturbation requires a particular sequence and length, both of which *Fos* ecRNA must contain but remains uncharacterized. The number of RNAs cells produce far exceeds the number of proteins that interact with these RNAs. In fact, a particular RNA may interact with multiple proteins and likewise proteins may interact with multiple RNA [61]. Therefore, it has been proposed that this promiscuity and malleability of RNA–protein interactions contributes to the complex network of these interactions in cells [61]. We propose that this is likely the case for DNMT3A-RNA interactions as it binds distinct RNA species in various biological contexts [1, 2, 12, 62, 63].

Our work complements the extensive cell biological studies that reveal complex crosstalk between DNA methylation, histone modification, and non-coding RNA [1–8, 11]. Clearly, our finding that non-coding RNA is a dominant form of DNMT3A regulation has implications on the extent to which distinct components of the epigenetic machinery contribute to the establishment of DNA methylation patterns. Furthermore, a major finding reported here is a model for RNA-mediated DNMT3A regulation in which RNA is produced locally (e.g., ecRNA, Fig. 6A, I–IV.) resulting in regulation of proximal DNMT3A enzymes through a non-specific binding site at the DNMT3A tetramer interface, coupled with the well-known rapid degradation of RNA [21, 64]. The relevance of this proposed mechanism to DNMT3B and DNMT1 remains to be determined. Short non-coding RNAs have been shown to inhibit DNMT1 activity by directly binding a distinct surface as DNA substrates [19]. Although our proposed mechanism for DNMT3A (Fig. 6A) is consistent with these findings, how the production of short non-coding RNAs contributes to the regulation of other DNMTs remains uncertain. Furthermore, while mechanisms to explain how long non-coding RNA regulates DNMTs, histone modifying enzymes, and transcription factors appear better supported by compelling data, how small non-coding RNA act in this capacity remains challenging [65, 66]. This derives in part because many models invoke mechanisms (R-loop or RNA–DNA triplex) that have yet to be demonstrated to directly regulate the target protein [20, 22, 23]. Finally, our implication of the tetramer interface as the site of RNA regulation, along with our prior identification of this interface in protein–protein interactions, highlights its potential for therapeutic intervention [3, 4, 41].

## Conclusion

Here we show that ecRNAs contribute to transcriptional regulation of their target genes on a single cell level. Furthermore, we provide evidence that this regulation may occur in the absence of ecRNA-DNA complexes and involve direct binding of ecRNA to the DNMT3A tetramer interface, a major site for protein–protein interactions. This mechanism of ecRNA-mediated regulation of DNMT3A catalysis expands our understanding of the role regulatory non-coding RNAs play in epigenetic regulation.

## Methods

### smFISH probe design

To quantify and localize *Fos* ecRNA and mRNA transcripts in situ, we designed Stellaris^®^ probe sets for fluorescent detection of *Fos* ecRNA (12 probes, conjugated to Quasar^®^ 570) and mRNA (30 probes, conjugated to Quasar^®^ 670). Probe sets consisted of multiple 14–20mer oligonucleotides targeting the same RNA molecule to optimize signal strength while minimizing background fluorescence. Target sequences of each probe set are provided in Table S1.

### Sample preparation and hybridization

*Day 1*: Primary neuronal cultures (~ 250,000 neurons per coverslip/well) were treated with KCl (25 mM) or vehicle treated for 1 h. After treatment cells were cross-linked with 3.7% formaldehyde (paraformaldehyde in 1X PBS) for 10 min at room temperature (21 °C) on a rocking platform. Wells were washed twice with PBS and permeabilized in 70% ethanol for at least 3 h at 4 °C. Wells were washed in Stellaris^®^ Wash Buffer A for 5 min at room temperature. Coverslips were transferred to a humidifying chamber and incubated with hybridization buffer (0.5 nM mRNA probe, 0.5 nM ecRNA probe) for 14 h at 37 °C. *Day 2:* Coverslips were washed twice in Stellaris^®^ Wash Buffer A for 60 min at 37 °C. After a 5 min wash in Stellaris^®^ Wash Buffer B at room temperature coverslips were mounted using ProLong^™^ antifade with DAPI for imaging.

### Quantification of expression

smRNA FISH results were quantified using StarSearch (http://rajlab.seas.upenn.edu/StarSearch/launch.html), which was developed by Marshall J. Levesque and Arjun Raj at the University of Pennsylvania to automatically count individual RNAs. mRNA and ecRNA detection involved two major steps. First, images for each probe set as well as a DAPI image were merged, and cells were outlined. Next, punctae detection was carried out and additional adjustment of thresholds was performed. The same threshold range was used for all images, and this analysis was performed blind to treatment group.

### Expression constructs

The plasmids used for expression of recombinant human proteins were as follows: pET28a-hDNMT3ACopt for DNMT3A full length (residues 1–912) [34], pET28a-hDNMT3A_catalytic_domain for wild type or mutants of DNMT3A catalytic domain (residues 634–912) [35], pTYB1–3L was used to express full-length human DNMT3L (residues 1–386) [36]. Mutations to the DNMT3A catalytic domain pET28a-hDNMT3A_catalytic_domain as a template) were generated as described in [4] and [41].

### Protein expression

DNMT3A (human full length and catalytic domain) and DNMT3L (human full length) were expressed in NiCo21(DE3) Competent *E. coli* cells (New England Biolabs). Following growth in LB media at 37 °C to an *A*_600 nm_ of 0.9 (DNMT3A full length) and 0.7 (DNMT3A catalytic domain and DNMT3L), induction occurred at room temperate (5 h for DNMT3A full length and catalytic domain, and 16 h for DNMT3L) with 1 mm IPTG (GoldBio). Cell pellets were harvested by centrifugation at 5000 g for 15 min and stored at − 80 °C.

### Protein purification

Proteins were purified as stated in [3] and [32]. Briefly, bacterial cell pellets were resuspended in lysis buffer (50 mM HEPES pH 7.8, 500 mM NaCl, 50 mM imidazole, 10% glycerol and 1 mM PMSF) and lysed by sonication. Cleared lysates (11,000 g for 1 h) were purified using ÄKTA Fast Protein Liquid Chromatography (FPLC) system with a 5 mL HisTrap HP nickel charged IMAC column (GE healthcare), which was previously equilibrated with loading buffer (50 mM HEPES pH 7.8, 500 mM NaCl, 50 mM imidazole, 10% glycerol). Resins were washed (50 mM HEPES pH 7.8, 500 mM NaCl, 75 mM imidazole, 10% glycerol) and an imidazole gradient was used to elute bound proteins (50 mM HEPES pH 7.8, 500 mM NaCl, 75–500 mM imidazole, 10% glycerol). Pooled fractions were desalted, concentrated into storage buffer (50 mM Tris–Cl, 200 mM NaCl, 1 mM EDTA, 20% (v/v) glycerol, pH 7.8, with 0.5 mM DTT) using a 15 mL Centrifugal Filter with a 10 K MWCO supplied by MilliporeSigma™, and stored at − 80 °C for later use. Protein concentrations were determined using 280 nm extinction coefficients (142,010 M^−1^ cm^−1^ for full length DNMT3A, 38,180 M^−1^ cm^−1^ for the catalytic domain of DNMT3A, 68380 M^−1^ cm^−1^ for full length DNMT3L) and reflect the oligomeric state in all experimental conditions (nM of tetramers for the full length and the catalytic domain of DNMT3A). A summary gel of the purified recombinant proteins used in this study is in the supporting information (Fig. S6).

### Computational modeling

The HDOCK docking server was employed to predict the surface on DNMT3A that interacts with *Fos-1* ecRNA using a DNMT3A (PDB: 4U7T, chain A) monomer and the *Fos-1* ecRNA sequence [34]. In the absence of any structures for *Fos-1* ecRNA or DNMT3A-RNA co-complexes, we relied on this approach as the HDOCK server utilizes a hybrid of global docking and a template-based modeling, which incorporates binding interface information from protein-RNA complexes in the PDB and has been previously employed for similar purposes [34, 42–44]. In the absence of a RNA crystal structure, like in the case of *Fos-1* ecRNA, the server initially searches the PDB to generate a template based predicted structure based on the highest sequence similarity, coverage, and resolution [34, 42]. To generate models, HDOCK relies on a Fast Fourier Transform (FFT)‐based docking algorithm to perform a global docking search to explore all possible binding modalities, which are then refined and scored by a knowledge‐based scoring function as well as backbone flexibility [34, 67].

### Methylation assays

In this study DNMT3A_FL refers to the full-length protein (912 amino acids) and DNMT3A_CD refers to the catalytic domain of DNMT3A (residues 634–912). DNA methylation reactions were carried out to assess the ability of DNMT3A to incorporate tritiated methyl groups from AdoMet onto distinct DNA substrates and experimental conditions. Assays were performed at 37 °C in a reaction buffer consisting of 50 mM KH_2_PO_4_/K_2_HPO_4_ (pH 7.8), 1 mM EDTA, 1 mM DTT, 0.2 mg/mL BSA, 20 mM NaCl with saturating AdoMet (15 μM). For the radiochemical assays, 50 µM ([^3^H] methyl-labeled: unlabeled, 1:10) AdoMet stocks were prepared from 32 mM unlabeled AdoMet (NEB) and [^3^H] methyl-labeled AdoMet (80 Ci/mmol; supplied by PerkinElmer) in 10 mM H_2_SO_4_. Reactions were carried out using two distinct substrates, *p15* gene promoter (54 CpG sites in a 749 bp helix) inserted into the pCpG^L^ plasmid (0 CpG sites in a 3467 bp helix) as well as Poly dI-dC (500CpG sites in a 1000 bp helix, obtained from Sigma Aldrich). We relied on *p15* as a substrate, in both nucleosome free and *p15*-pCpG^L^ assembled into polynucleosomes, as *p15* is a well-established target of DNMT3A that has been previously characterized biochemically and Poly dI-dC due to the increased activity of DNMT3A in this substrate, which allows a higher level of detection to changes in enzymatic activity [4]. Reactions were quenched by mixing aliquots taken from a larger reaction with 0.1% SDS (1:1) and spotted onto Hybond-XL membranes (GE healthcare). Samples were then washed, dried, counted using a Beckman LS 6000 liquid scintillation Counter and radioactive counts were converted to nM product as described in [68, 69]. Backgrounds consisting of protein in reaction buffer with labelled and unlabelled AdoMet (1:10) were subtracted from all data shown. Reactions with RNAs (Fig. S7) *Fos-1* (5′-GGGGACACGCCCUCUGUUCCCUUAU-3′), *Fos-2* (5′-GUCUGUGCACCGUGUGCAUAUACAG-3′) or non-specific (5′-CGACCGCCUACUGAAAGAGGGC-3′) were initiated by the addition of RNA pre-mixed with distinct DNA substrates (Poly dI-dC, nucleosomal or non-nucleosomal *p15*) as previously described in [2]. The RNAs used in this study were purchased from IDT and RNase-free HPLC purified. Where indicated, fold inhibition refers to 1 − (product formed with RNA/product formed without RNA) whereas fold change refers to data normalized to the activity of reactions consisting of DNMT3A only. In reactions consisting of additional modulators of DNMT3A activity (synthetic H3K4me0 peptide or DNMT3L), DNMT3A was pre-incubated with H3K4me0 or DNMT3L in reaction buffer for 1 h at 37 °C prior to being initiated. Synthetic peptides derived from human Histone H3.1 (N-ARTKQTARKSTGGKAPRKQLA-C) were supplied by Active Motif [70]. In vitro reconstitution of nucleosome core particles extracted from HeLa cells onto *p15*-pCpG^L^ DNA was performed with a salt gradient [71, 72]. For the high-salt histone extraction, HeLa cells (10^7^) were harvested (300 g for 10 min) and re-suspended in hypotonic lysis buffer (10 mM Tris–Cl pH 8.0, 1 mM KCl, 1.5 mM MgCl_2_ and 1 mM DTT). The nuclei were pelleted (10,000 g for 10 min at 4 °C), re-suspended in extraction buffer (10 mM HEPES pH 7.9, 10 mM KCl, 1.5 mM MgCl_2_, 0.34 M sucrose, 10% glycerol) and lysed in no-salt buffer (3 mM EDTA, 0.2 mM EGTA) by vortexing for 1 min (10 s on and off). The chromatin was pelleted (6500 g for 5 min), re-suspended in high-salt solubilization buffer (50 mM Tris–Cl pH 8.0, 2.5 M NaCl) by vortexing for 2 min. DNA was pelleted (16,000 g for 10 min) and the supernatant was transferred to a dialysis cassette (3500 MWCO) against a no-salt dialysis buffer (10 mM Tris–Cl pH 8.0) for 2 h at at 4 °C and stored at − 80 °C [70]. Nucleosome core particles were reconstituted by salt gradient deposition, in which DNA and histone extracts were mixed in 2 M NaCl (30 min at 4 °C) followed by the addition of dilution buffer (10 mM Tris–HCl, pH 7.6; 1 h at at 4 °C). Dilution buffer was then added (1 h incubation at 4 °C per dilution) to obtain the following concentrations of NaCl (M): 0.8, 0.6, 0.2 and 0.1 [71].

### Fluorescence anisotropy

Fluorescence anisotropy measurements were obtained using a Horiba Fluoromax fluorescence spectrophotometer equipped with excitation and emission polarizers (excitation: 485 nm, emission 535 nm) following a 5-min incubation at room temperature. Reactions involving DNMT3A (or DNMT3A-DNMT3L) complexes with FAM-labelled DNA or H3K4me0 peptide binding to *Fos-1* ecRNA were carried out in the following buffer: 50 mM KH_2_PO_4_/K_2_HPO_4_ (pH 7.8), 1 mM EDTA, 1 mM DTT, 0.2 mg/ml BSA, 20 mM NaCl with 50 μm Sinefungin. The DNA substrate (Gcbox30) consisted of a duplex with a fluorescein (6-FAM) label on the 5′ end of the top strand (5′/6 FAM/TGGATATCTAGGGGCGCTATGATATCT-3′; the recognition site for DNMT3A is underlined) [41]. Peptide binding experiments consisted of H3K4me0 (residues 1–21) with a FAM-NHS label on the N-terminus [32]. Reactions involving a segment of the human *Fos* gene (NCBI Gene ID 2353, 3′- 500 nucleotide duplex DNA) and 5′ FAM-6-labeled RNA were carried out in triplex buffer (20 mM Tris–HCl pH 7.4, 5 mM EDTA, 25 mM NaCl, 10 mM MgCl_2_, 200 μM DTT) and incubated for 30 min at 37 °C for 30 min prior to measuring the fluorescence anisotropy. The 5′ 6-FAM-labeled RNAs employed were an 18-mer positive control RNA designed to complex with the *Fos* gene (NCBI Gene ID 2353, 3′- 500 nucleotide duplex DNA) or *Fos-1* ecRNA.

## Supplementary Information

Supplementary Material 1. This article contains the following supporting information:Fig. S1. Functional characterization of RNA-mediated inhibition of DNMT3A_CD^WT^ activity and selectivity of *Fos-1* ecRNA for human DNMTs. Fig. S2. Computational models predict *Fos-1* ecRNA binds at the tetramer interface of DNMT3A. Fig. S3. Modulation of DNMT3A_CD^WT^ enzymatic activity by *Fos-1* ecRNA is dominant in the presence of DNMT3L. Fig. S4. Binding of Full-length DNMT3A^WT^ to FAM-labeled H3K4me0 peptides. Fig. S5. Excess *Fos-1 *ecRNA inhibits Full-length DNMT3A^WT^ activity with polynucleosomes as substrates. Fig. S6. Summary gel of proteins used in this study. Fig. S7. Predicted secondary structures of RNAs used in this study. Fig. S7. Interactions between DNMT3A R771 and DNMT3L at the tetramer interface. Table S1. Target sequences and dyes of probes used in Single Molecule RNA FISH.

## Data Availability

No datasets were generated or analysed during the current study.

## Abbreviations

DNMT3A: DNA methyltransferase 3A
ecRNAs: Extra-coding RNAs
AML: Acute Myeloid Leukemia
TCGA: The Cancer Genome Atlas
UCEC: Uterine corpus endometrial carcinoma
smFISH: In situ Hybridization
CDKN2B or p15: Cyclin-dependent kinase inhibitor 2B
ADD: Atrx-Dnmt3-Dnmt3l ADD
