# Supplementary material for: Mechanism of non-coding RNA regulation of DNMT3A
Source: Epigenetics Chromatin. 2025 Mar 28;18:15. doi: 10.1186/s13072-025-00574-w (PMC11951571; doi:10.1186/s13072-025-00574-w)

**Mechanism of non-coding RNA regulation of DNMT3A**

**Jonathan E. Sandoval*^1, 2^, Nancy V.N. Carullo^3^, Aaron J. Salisbury^3^, Jeremy J. Day^3^ and Norbert O. Reich*^2^**

From the ^1^Department of Molecular, Cellular and Developmental Biology, University of California, Santa Barbara, California 93106-9510. ^2*^Department of Chemistry and Biochemistry, University of California, Santa Barbara, California 93106-9510. ^3^Department of Neurobiology, University of Alabama at Birmingham, Birmingham, AL 35294.

*To whom correspondence should be addressed: Norbert O. Reich: Department of Chemistry and Biochemistry, University of California, Santa Barbara, California 93106-9510; [reich@chem.ucsb.edu](mailto:reich@chem.ucsb.edu); Tel. (805) 893-8368.

This PDF includes the following:

1. Fig. S1. Functional characterization of RNA-mediated inhibition of DNMT3A_CD^WT^ activity and selectivity of *Fos-1* ecRNA for human DNMTs.
2. Fig. S2. Computational models predict *Fos-1* ecRNA binds at the tetramer interface of DNMT3A.
3. Fig. S3. Modulation of DNMT3A_CD^WT^ enzymatic activity by *Fos-1* ecRNA is dominant in the presence of DNMT3L.
4. Fig. S4. Binding of Full-length DNMT3A^WT^ to FAM-labeled H3K4me0 peptides.
5. Fig. S5. Excess *Fos-1* ecRNA inhibits Full-length DNMT3A^WT^ activity with polynucleosomes as substrates.
6. Fig. S6. Summary gel of proteins used in this study.
7. Fig. S7. Predicted secondary structures of RNAs used in this study.
8. Fig. S7. Interactions between DNMT3A R771 and DNMT3L at the tetramer interface.
9. Table S1. Target sequences and dyes of probes used in Single Molecule RNA FISH.


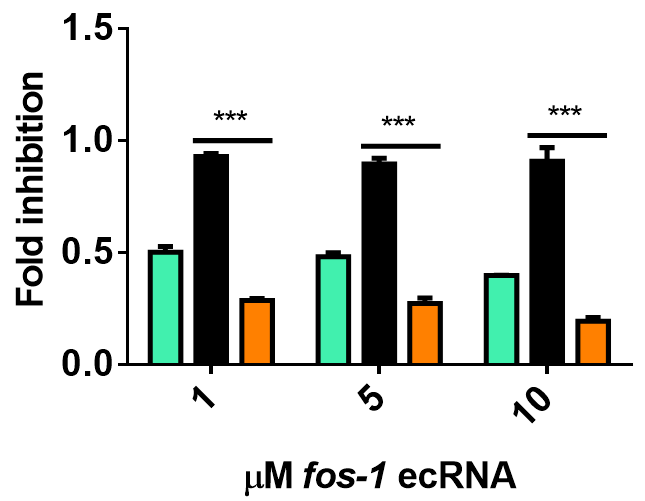


A.

B.

C.


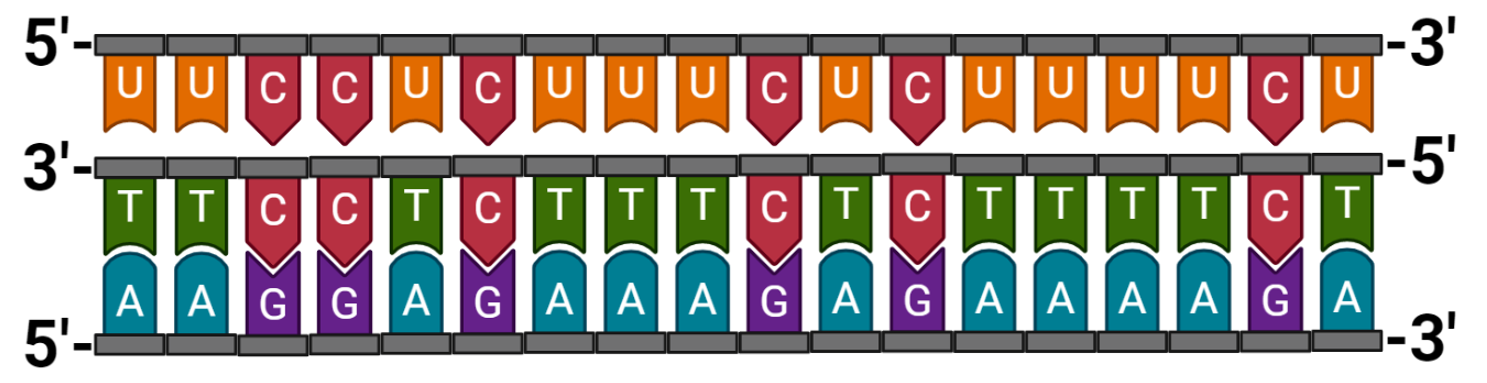

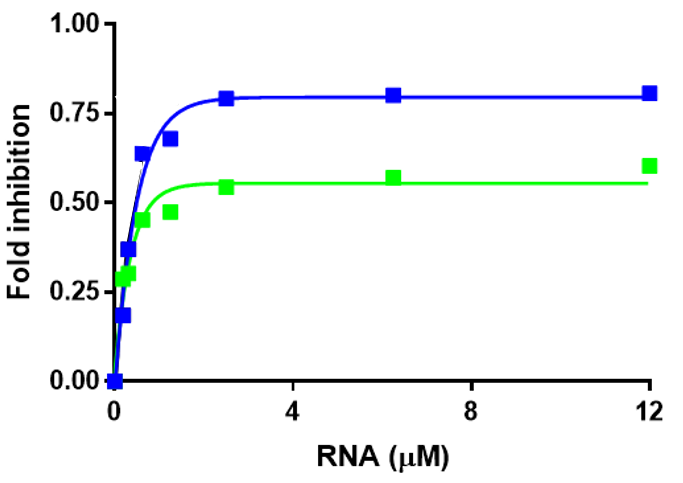


**Fig. S1. Functional characterization of RNA-mediated inhibition of DNMT3A_CD^WT^ activity and selectivity of *Fos-1* ecRNA for human DNMTs.** (A.) sequences for oligonucleotide binding assays. The design relies on canonical principles for RNA-DNA triplex formation with Polypurine (bottom strand) and Polypyrimidine (top strand) DNA strands found within the *Fos* gene body (NCBI Gene ID 2353, 3’- 500 nucleotide duplex DNA). (B.) The addition of a pre-mixture of Poly dI-dC (5 µM) and increasing concentrations of *Fos-1* ecRNA inhibits reactions consisting of DNMT3A_CD (**■**, 150 nM) and DNMT1 (**■**, 133 U/mL) but not M. HhaI (**■**, 150 nM). (C.) Inhibition curves for *Fos-2* (**■**) and *Fos-1* (**■**) ecRNAs with DNMT3A_CD^WT^. Data in (B.) and (C.) reflect the mean and standard deviation of 3 experiments; In (B.), a one-way analysis of variance was used to compare the values of each sample with increasing *Fos-1* ecRNA (***, *p* < 0.001; ns, *p* > 0.05).


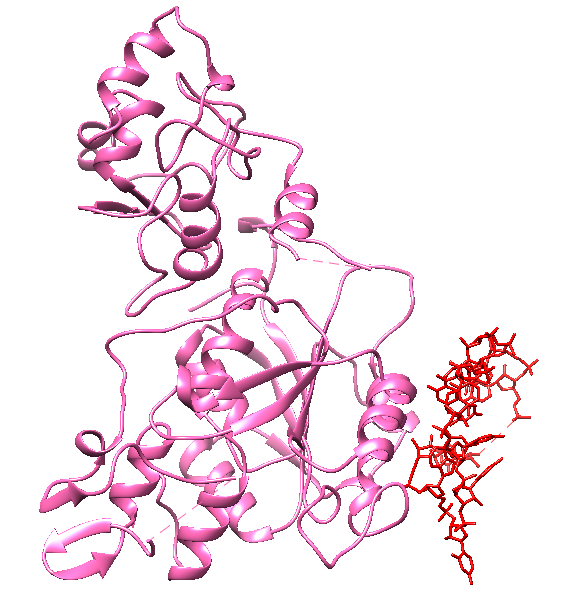

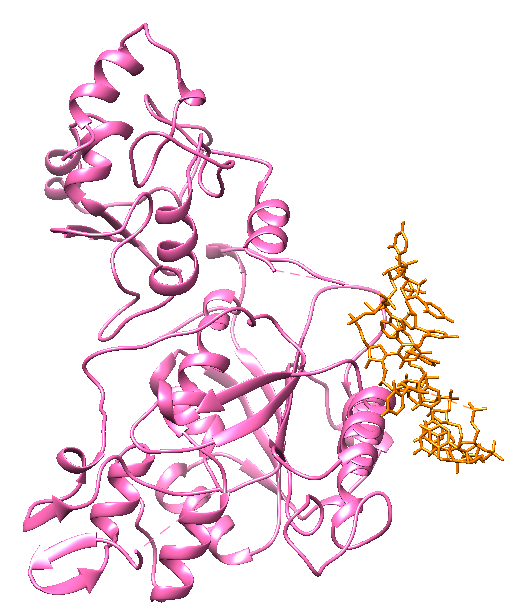

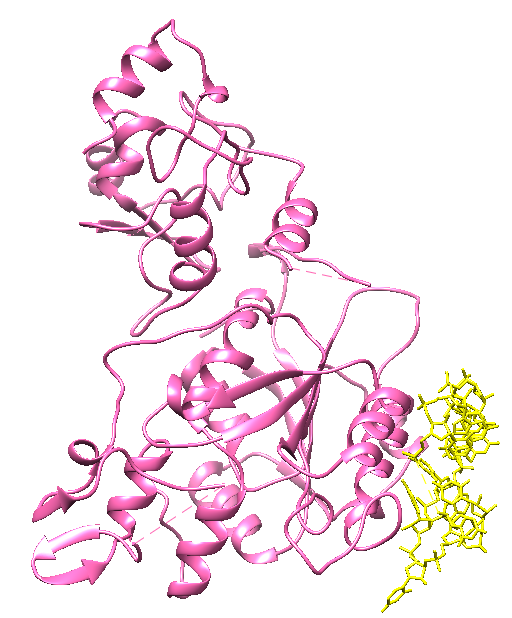

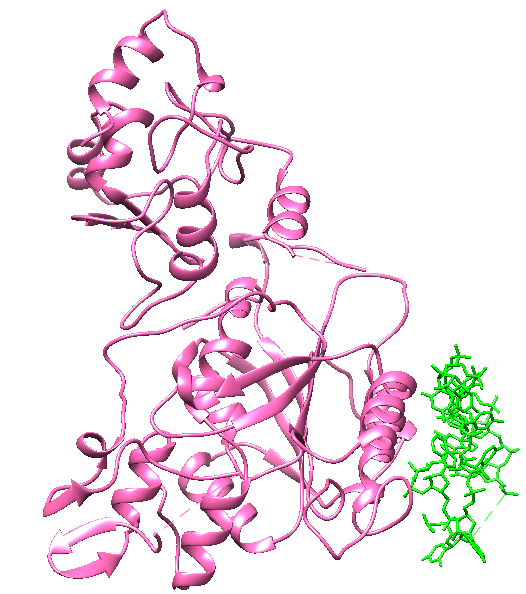

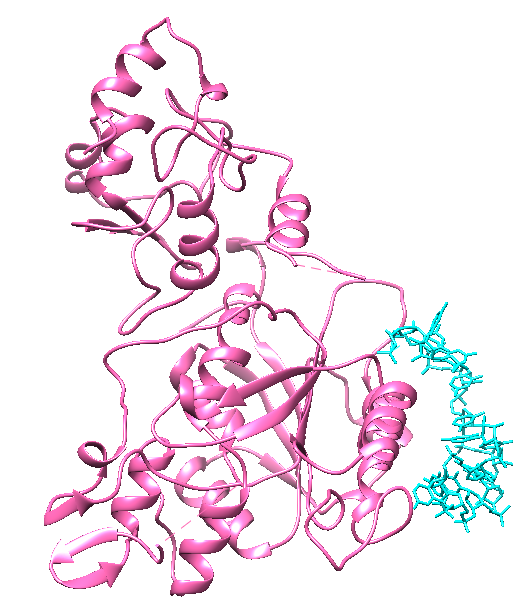

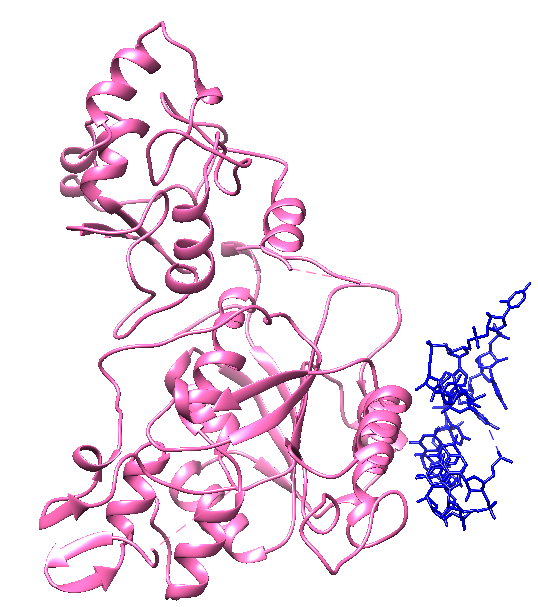

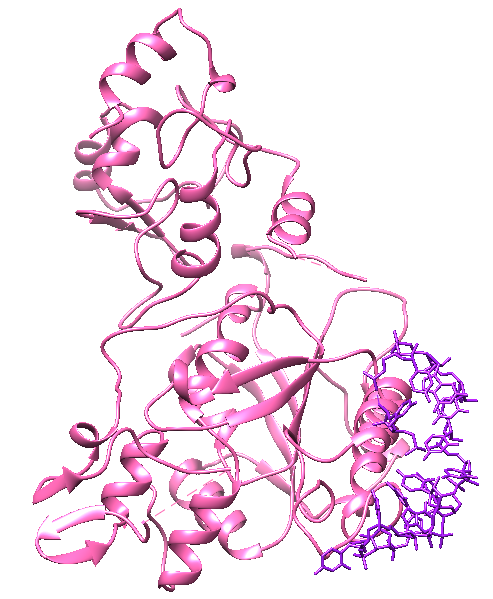

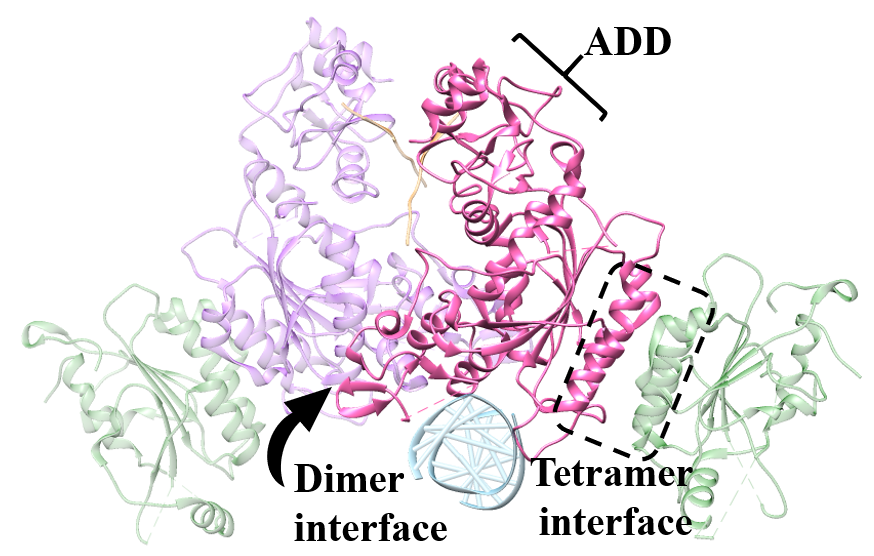


**II.**

**III.**

**IV.**

**V.**

**VI.**

**VII.**

**VIII.**

**I.**

**Fig. S2. Computational models predict *Fos-1* ecRNA binds at the tetramer interface of DNMT3A.** (I.) Crystal structure of a DNMT3A (■ and ■; residues 468-912) in complex with DNMT3L (■; residues 171-379) and H3 tail peptides (■; 1-21) (adapted from PDB 4U7T). (II.-VIII.) computational models of DNMT3A monomers in complex with *Fos-1* ecRNA.


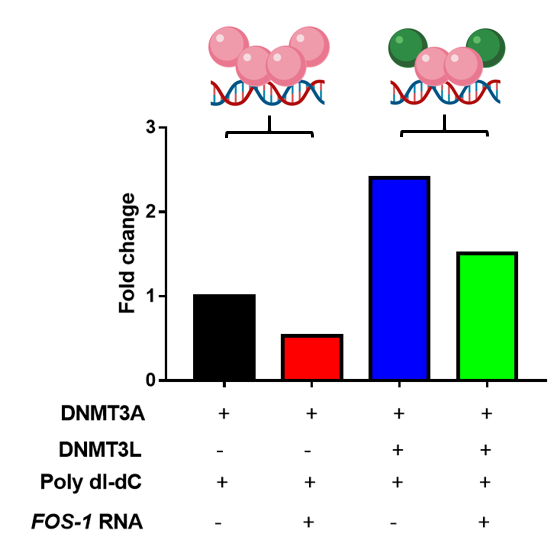


**Fig. S3. Modulation of DNMT3A_CD^WT^ enzymatic activity by *Fos-1* ecRNA is dominant in the presence of DNMT3L.** *Fos-1* ecRNA inhibits enzymatic activity in reactions consisting of DNMT3A_CD^WT^ homotetramers (**■**) or DNMT3A_CD^WT^**-**DNMT3L heterotetramers (**■**). Reactions consisted of proteins at 150 nM (1:1 to DNMT3A_CD^WT^**-**tetramer) and were initiated by the addition of a pre-mixture of *Fos-1* ecRNA (1 μM) and Poly dI-dC (5 μM).


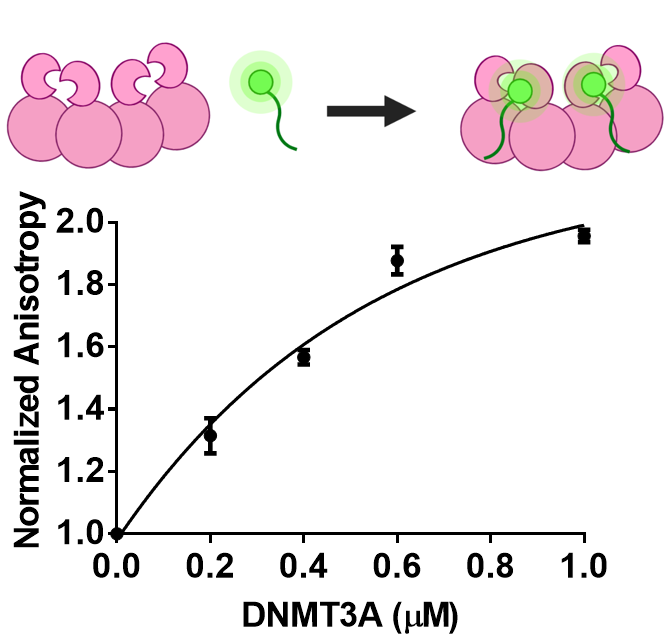


**Fig. S4. Binding of Full-length DNMT3A^WT^ to FAM-labeled H3K4me0 peptides.** Increasing amounts of full-length DNMT3A^WT^ leads to a concomitant increase in the fluorescence anisotropy of 10 nM of 5′ FAM-labeled H3K4me0 (residues 1-21).


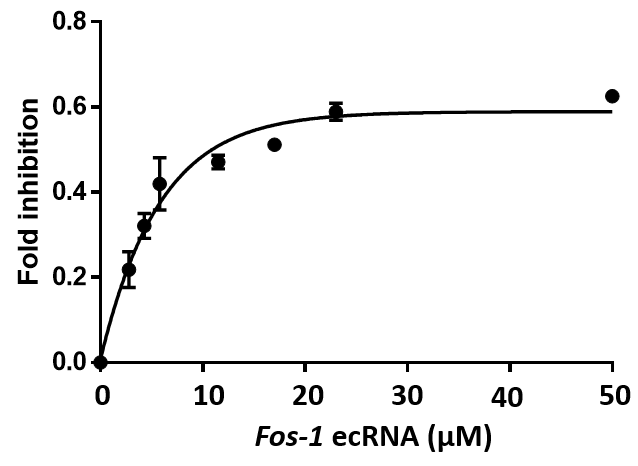

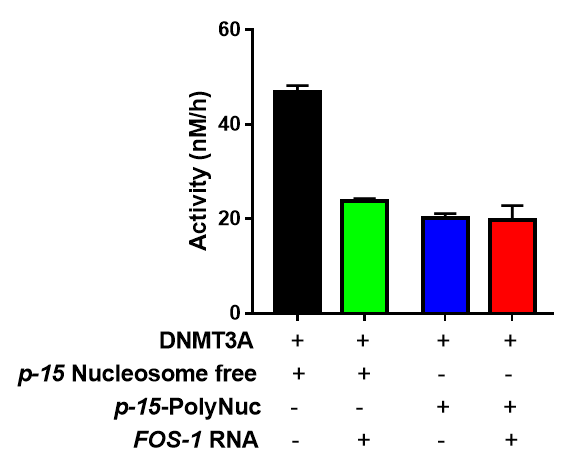


A.

B.

**Fig. S5. Excess *Fos-1* ecRNA inhibits Full-length DNMT3A^WT^ activity with polynucleosomes as substrates.** *Fos-1* ecRNA (1 μM) inhibits the enzymatic activity of full-length DNMT3A^WT^ (150 nM) with nucleosome*-*free p15 as a substrate but not with the use of p15 assembled into polynucleosomes (A.). Reactions consisted of 14 μM *p15* substrate (Nucleosome free or polynucleosomes) and were initiated by the addition of *p15* substrate (Nucleosome free or polynucleosomes) or a pre-mixture of *Fos-1* ecRNA with *p15* (Nucleosome free or polynucleosomes). (B.) Excess concentration of *Fos-1* ecRNA inhibits full-length DNMT3A^WT^ (150 nM) with p15 polynucleosomes (14 μM) as substrates.


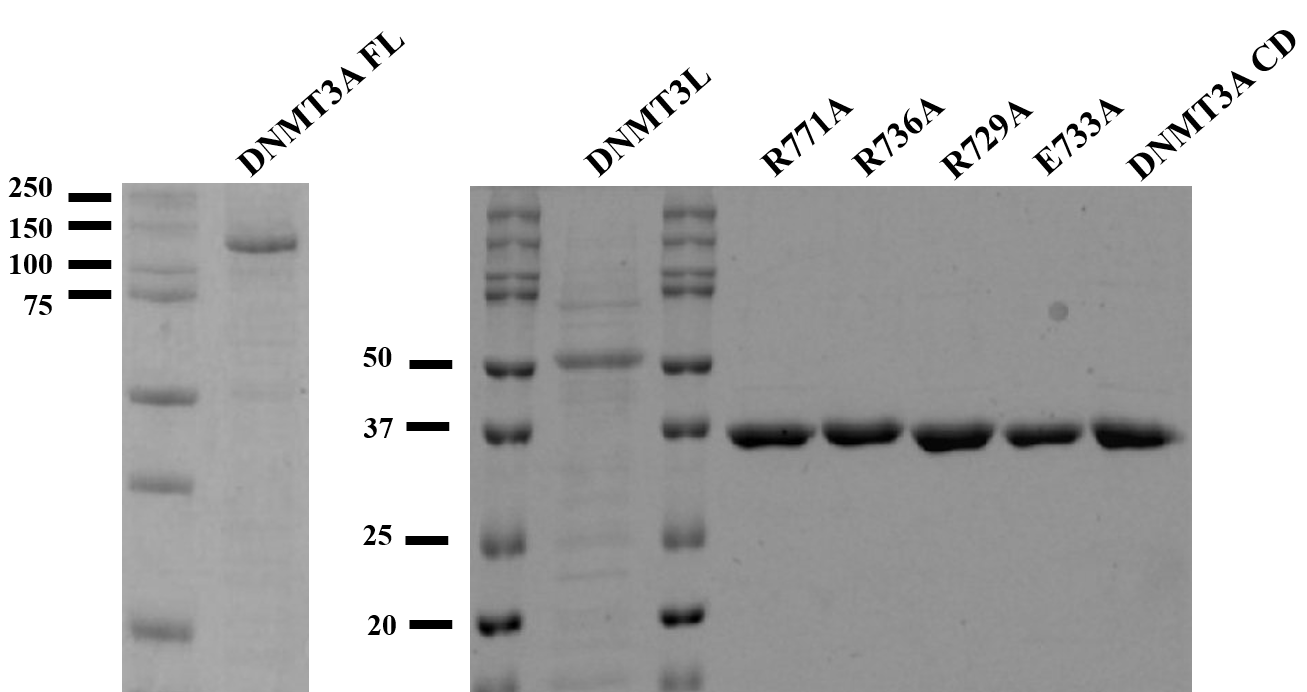


**Fig. S6. Summary gel of proteins used in this study.** Proteins were run (150 Volts) on a 12% SDS-PAGE gel in 1X SDS electrophoresis buffer. Precision Plus Protein Dual Color (BIO-RAD) was used as a standard.


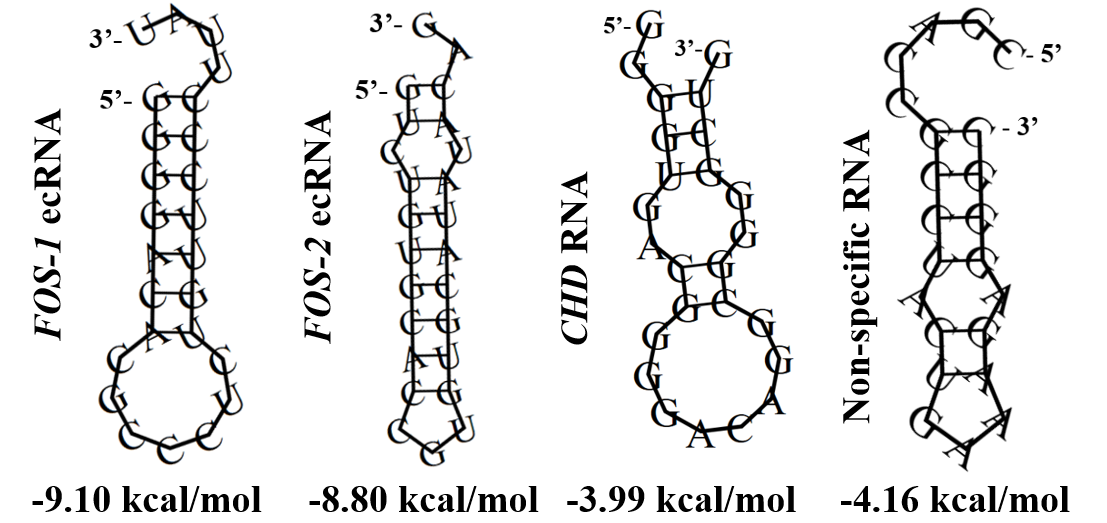

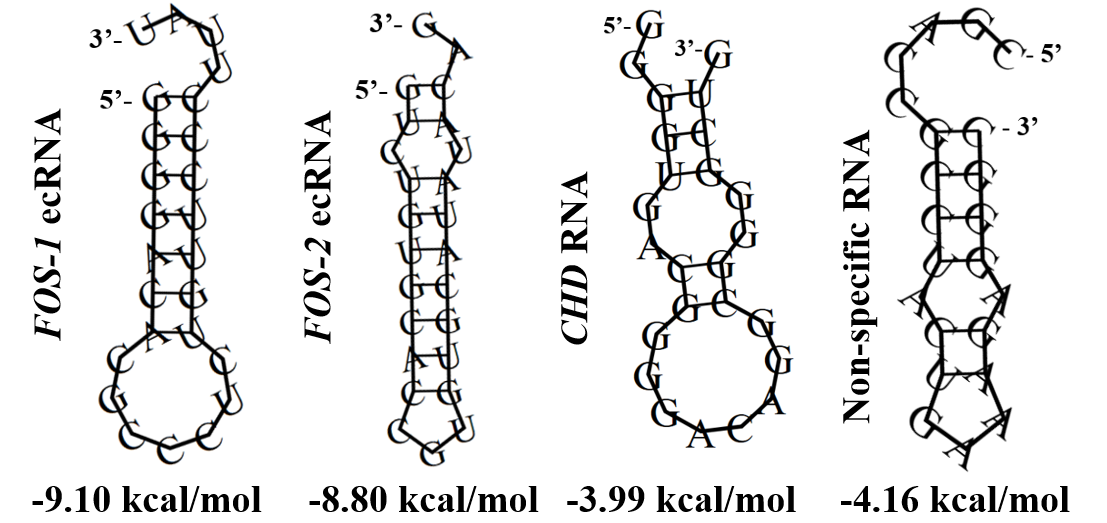


**Fig. S7. Predicted secondary structures of RNAs used in this study.** Predicted structures were generated using the RNAfold WebServer from the Institute for Theoretical Chemistry at the University of Vienna.


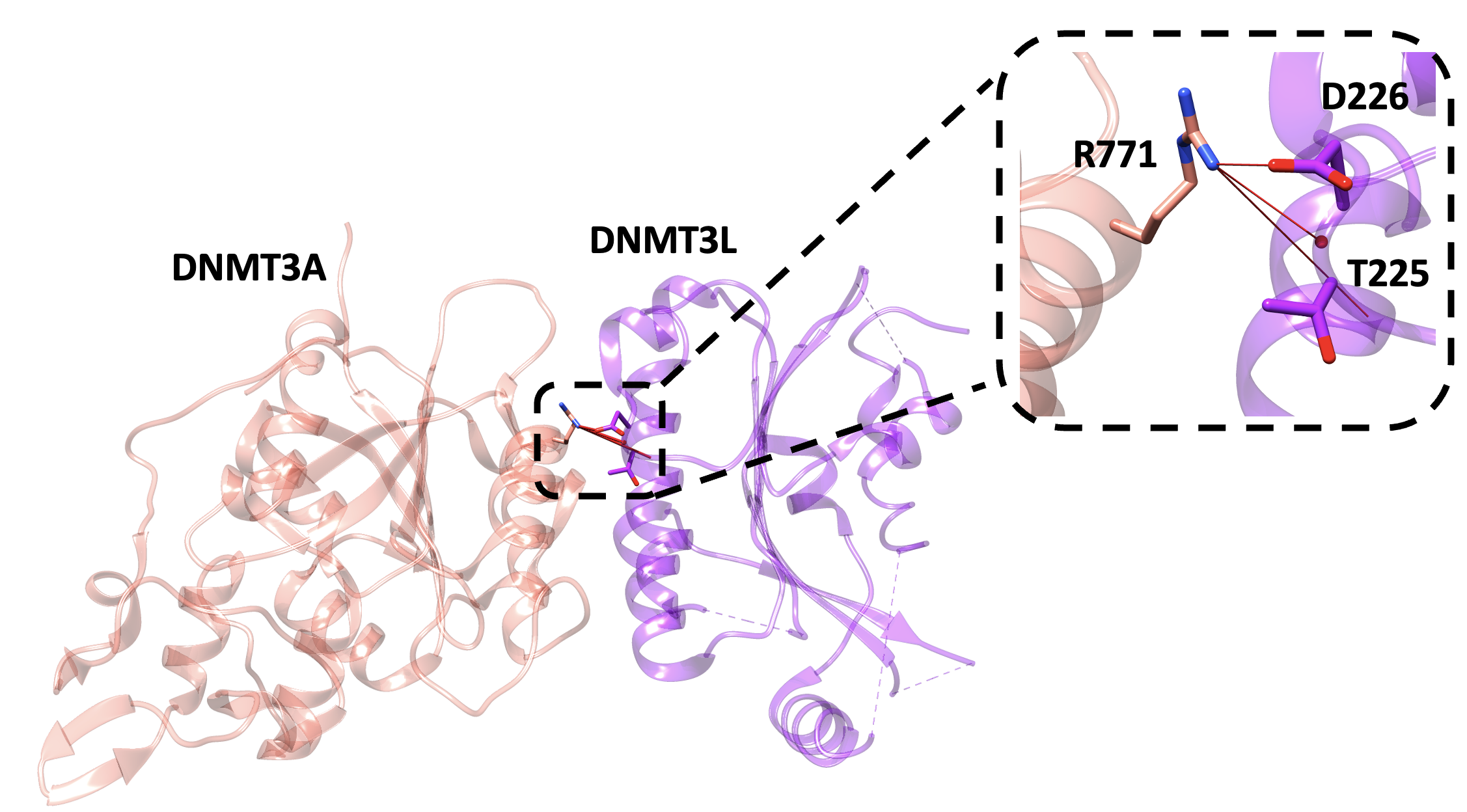


**Fig. S8. Interactions between DNMT3A R771 and DNMT3L at the tetramer interface.** Crystal structure of a DNMT3A (■ ; residues 468-912) in complex with DNMT3L (■; residues 171-379) (adapted from PDB 5YX2). Red lines depict interactions between R771 and DNMT3L residues < 5 Å.

**Table S1. Target sequences and dyes of probes used in Single Molecule RNA FISH.**


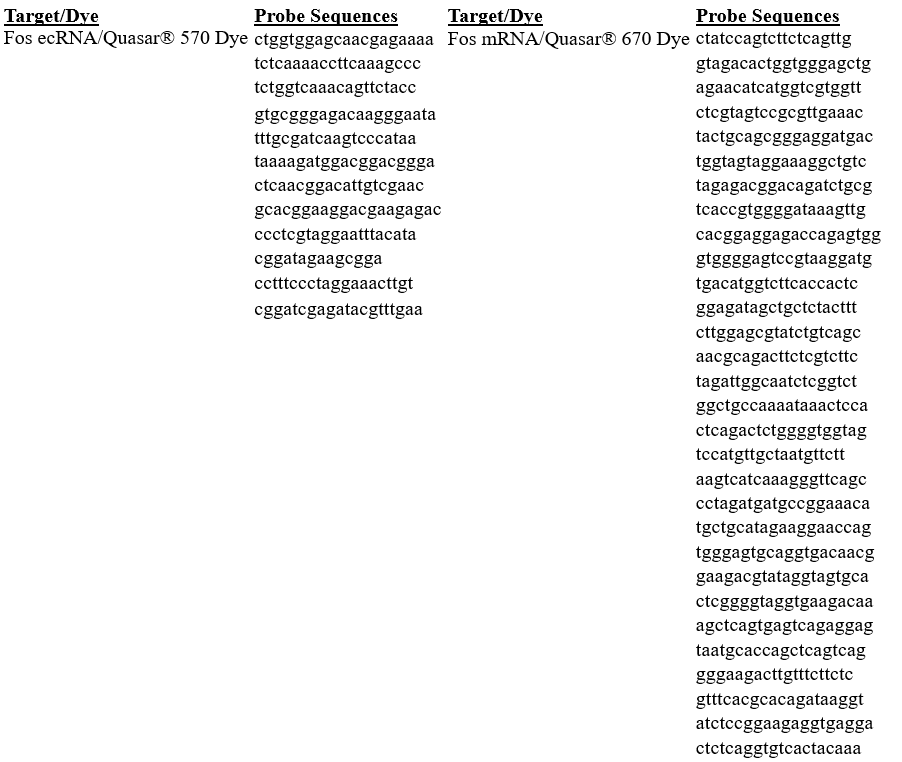

Supplement: Supplementary file 1 — Supplementary Material 1. This article contains the following supporting information:Fig. S1. Functional characterization of RNA-mediated inhibition of DNMT3A_CDWT activity and selectivity of Fos-1 ecRNA for human DNMTs. Fig. S2. Computational models predict Fos-1 ecRNA binds at the tetramer interface of DNMT3A. Fig. S3. Modulation of DNMT3A_CDWT enzymatic activity by Fos-1 ecRNA is dominant in the presence of DNMT3L. Fig. S4. Binding of Full-length DNMT3AWT to FAM-labeled H3K4me0 peptides. Fig. S5. Excess Fos-1 ecRNA inhibits Full-length DNMT3AWT activity with polynucleosomes as substrates. Fig. S6. Summary gel of proteins used in this study. Fig. S7. Predicted secondary structures of RNAs used in this study. Fig. S7. Interactions between DNMT3A R771 and DNMT3L at the tetramer interface. Table S1. Target sequences and dyes of probes used in Single Molecule RNA FISH. [file 13072_2025_574_MOESM1_ESM.docx]
